# TMEM52B suppression promotes cancer cell survival and invasion through modulating E-cadherin stability and EGFR activity

**DOI:** 10.1186/s13046-021-01828-7

**Published:** 2021-03-01

**Authors:** Yunhee Lee, Dongjoon Ko, Junghwa Yoon, Younghoon Lee, Semi Kim

**Affiliations:** 1 Immunotherapy Research Center, Korea Research Institute of Bioscience and Biotechnology, Daejon, South Korea; 2 Department of Chemistry, Korea Advanced Institute of Science and Technology, Daejon, South Korea; 3 Department of Functional Genomics, Korea University of Science and Technology, Daejon, South Korea

**Keywords:** TMEM52B, Cell survival, Invasion, EGFR, E-cadherin, Prognostic marker

## Abstract

**Background:**

TMEM52B is a novel gene broadly expressed in a variety of normal human tissues. However, the biological function of TMEM52B expression in cancer is largely unknown.

**Methods:**

The effects of TMEM52B on tumor growth and metastasis were investigated in vitro and in vivo, and the underlying biological and molecular mechanisms involved in this process were evaluated. Clinical datasets from KmPlotter and The Cancer Genome Atlas (TCGA) were analyzed in relation to TMEM52B expression and function.

**Results:**

Suppression of TMEM52B in colon cancer cells promoted cancer cell epithelial-mesenchymal transition (EMT), invasion, and survival in vitro. Similarly, in vivo studies showed increased tumor growth and circulating tumor cell survival (early metastasis). ERK1/2, JNK, and AKT signaling pathways were involved in TMEM52B suppression-induced invasiveness and cell survival. TMEM52B suppression promoted activation and internalization of epidermal growth factor receptor (EGFR) with enhanced downstream signaling activity, leading to enhanced cell survival and invasion. In addition, TMEM52B suppression reduced E-cadherin stability, likely due to a reduced association between it and E-cadherin, which led to enhanced β-catenin transcriptional activity. Concomitantly, TMEM52B suppression promoted generation of soluble E-cadherin fragments, contributing to the activation of EGFR. Clinical data showed that high TMEM52B expression correlated with increased patient survival in multiple types of cancer, including breast, lung, kidney, and rectal cancers, and suggested a correlation between TMEM52B and E-cadherin.

**Conclusions:**

These findings suggest that TMEM52B is a novel modulator of the interplay between E-cadherin and EGFR. It is possible that TMEM52B functions as a tumor-suppressor that could potentially be used as a novel prognostic marker for cancer.

**Supplementary Information:**

The online version contains supplementary material available at 10.1186/s13046-021-01828-7.

## Background

Metastasis is the leading cause of cancer-related deaths in most cancer types. A metastatic cascade has several elements, including local invasion, intravasation, cancer cell survival in the circulation, extravasation, micrometastasis, and metastatic colonization [1]. Early in cancer metastasis, epithelial tumor cells are activated to invade surrounding stromal tissue through the epithelial-mesenchymal transition (EMT) process. EMT also plays important roles in next steps such as intravasation, cancer cell survival in the circulation, and micrometastasis. During EMT, cells undergo morphological and molecular changes to acquire a more mesenchymal phenotype [2–5]. A hallmark of EMT is the functional loss of E-cadherin, a well-known metastasis suppressor [5]. E-cadherin mediates cell-cell adhesion through Ca^2+^-dependent homophilic interactions, thereby suppressing cell migration and dissemination. In addition, E-cadherin anchors β-catenin to the cell membrane as part of a complex to prevent β-catenin from entering the nucleus and inducing the expression of EMT-related genes, thereby inhibiting EMT and suppressing cancer metastasis [5]. Downregulation of E-cadherin is usually mediated by E-cadherin transcriptional repressors/EMT-inducing transcription factors and is also mediated at the post-translational level [5, 6]. Furthermore, soluble E-cadherin fragment, formed by cleavage of E-cadherin, has been reported to contribute to tumor progression by promoting invasion and metastasis [6].

Epidermal growth factor receptor (EGFR) plays key roles in essential cellular functions, including proliferation, migration, and survival. EGFR is also an important driver of tumorigenesis mostly in glioblastoma, as well as lung, breast, and colon cancers. Inappropriate activation of EGFR through gene amplification, mutation, abnormal protein overexpression, ligand overproduction, and dysregulated intracellular trafficking plays an important role in cancers [7]. EGF-to-EGFR binding at the cell surface induces receptor dimerization and activation of its tyrosine kinase domain and downstream signaling events, including both PI3K/AKT and RAS/RAF/MEK pathways, which are critical for controlling proliferation, migration, and survival. Concomitantly, the receptor-ligand complexes are also internalized into endosomal compartments and routed to lysosomes for degradation, thereby terminating receptor activation and downstream signaling, or are sorted for recycling. However, internalized EGF-EGFR complexes can continue signaling within early endosomes and multivesicular bodies until their disassembly and degradation. Therefore, EGFR endocytosis can result in either positive or negative effects on signaling and tumorigenesis [7, 8].

Transmembrane protein 52B (TMEM52B; gene ID, 120939; chromosome location, 12p13.2; also termed C12orf59 or FLJ31166) was first cloned in 2002 [9] and has been recently shown to be expressed at the mRNA level in kidney (https://www.genecards.org/). TMEM52B mRNA isoform 1 (NM_153022; 2789 bp) encodes a 163 amino acid protein, and isoform 2 (NM_001079815; 2563 bp) differs from it in the 5′ UTR, containing an alternate exon in the 5′ coding region. Isoform 2 also initiates translation from an alternate upstream start codon, and encodes a distinct N-terminal, 183 amino acid protein. The TMEM52B gene shares homology with other species, including mouse, rat, frog, and chicken.

Recently, broad TMEM52B expression was reported in normal human tissues, with high expression in kidney; however, its expression in a panel of cancer cell lines was not detectable [10]. In that study, decreased TMEM52B expression was correlated with poor survival and von Hippel-Lindau mutations in renal cell carcinoma patients [10], suggesting that decreased TMEM52B expression may promote renal carcinogenesis.

However, general biological or cancer-biology functions for TMEM52B expression have yet to be determined. Here we report that suppression of TMEM52B in cancer cells increases cell survival and invasion in vitro, and enhances tumor growth and early metastasis in vivo. TMEM52B suppression enhanced EGFR activation and the downstream MAPK and AKT signaling pathways, leading to cancer cell invasion and survival. TMEM52B suppression mediated the shedding of extracellular E-cadherin and contributed to EGFR activation. These results suggest that TMEM52B is a novel modulator of EGFR and E-cadherin, with tumor/metastasis-suppressing activity. Thus, a reduction in TMEM52B expression may contribute to tumor progression.

## Materials and methods

### Cell lines

Human embryonic kidney 293E (HEK293E), SW480, Colo205, HCT-15, HCT-116, HT-29, SW620, Caco-2 (colon cancer), KATOIII, MKN28, MKN45 (gastric cancer), SKOV3, and IGROV1 (ovarian cancer) cell lines were purchased from the American Type Culture Collection (ATCC; Manassas, VA, USA). SW480sub cell was a subclone isolated from SW480 cells in our laboratory, which displayed elevated cell-matrix adhesion capacity compared to SW480 cells. SNU-216, SNU-638, SNU-668, and SNU-719 (gastric cancer) cell lines were purchased from the Korean Cell Line Bank (KCLB; Seoul, Korea). HEK293E cells were maintained in DMEM with 10% fetal bovine serum (FBS) at 37 °C in 5% CO_2_. SW480, HCT-116, HT-29, Colo205, HCT-15, SW620, KATOIII, MKN28, MKN45, SKOV3, and IGROV1 cells were maintained in RPMI1640 with 10% FBS at 37 °C in 5% CO_2_. Caco-2 cells were maintained in MEM with 10% FBS at 37 °C in 5% CO_2_. Cell were checked for mycoplasma and their identities were confirmed using STR-PCR analysis.

### Plasmid and cDNA constructs

cDNAs encoding two isoforms of wild-type full-length human TMEM52B were amplified by PCR with primer set (Forward primers for both vectors are 5′- AACATCTCGAGGCCGCCATGTCGTGGCGGCCTC-3′ for isoform 1 and 5′- AACATCTCGAGGCCGCCATGGGAGTCCGAGTTCAT-3′ for isoform 2. Reverse primers for both isoforms are 5′-AATTCAAGCTTGTTCCAAGAGTCAACTATTCG-3′ for pcDNA3.1-myc and 5′-CTTCGAATTCTGTTCCAAGAGTCAACTATTCG-3′ for pEGFP-N1) using cDNA from SW480 cells. The PCR product was digested with XhoI/HindIII and XhoI/EcoRI and then cloned into the expression vectors pcDNA3.1-myc and pEGFP-N1, respectively. The pcDNA3.1-myc was generated by inserting stop codon after myc tag into pcDNA3.1-myc-his vector. For flow cytometry analysis, cDNA fragments encoding two isoforms of TMEM52B (without the putative signal peptide sequence (amino acid residues 1–24) in the case of isoform 2) with a myc tag at the N-terminus were amplified by PCR and then subcloned into pcDNA3.1 vector/XhoI-HindIII. PCR primer set used was as follows; forward primers are 5′-CTCGAGGCCGCCATGGAACAAAAACTCATCTCAGAAGAGGATCTGTCGTGGCGGCCTCAGCCC-3′ for isoform 1 and 5′-CTCGAGGCCGCCATGGAACAAAAACTCATCTCAGAAGAGGATCTGGAGGAAAACTGTGGTAAT-3′ for isoform 2 and reverse primer for both isoforms is 5′-AAGCTTTCAGTTCCAAGAGTCAACTATTCG-3′.

### Reverse transcription-polymerase chain reaction (PCR)

Total RNA was isolated using TRIzol (Invitrogen, Carlsbad, CA), and cDNA was synthesized using reverse transcriptase (Bioneer, Daejon, Korea). Real-time quantitative PCR was performed using SYBR Green (PKT, Seoul, Korea) on a Rotor-Gene 6000 real-time rotary analyzer (Corbett, San Francisco, CA) with TMEM52B (both isoforms)-specific primers (5′- AGTGACCGTCATTGCTTTCG-3′ and 5′-GGCCAAACACCGACTGCAG − 3′), isoform 1-specific primers (5′-TGGTCAGGCAACAGGATGAA-3′ and 5′-ACAGCAGCAGCGGAAGCAC-3′), isoform 2-specific primers (5′- GTGGTCAGGCAATGTTCAGG-3′ and 5′-ACAGCAGCAGCGGAAGCAC-3′), and GAPDH-specific primers (5′-CATGACCACAGTCCATGCCAT-3′ and 5′-AAGGCCATGCCAGTGAGCTTC-3′) with an annealing temperature of 61 °C.

### Transfection of siRNAs, shRNA vectors, and expression vectors

Cells were transfected with siRNA specific to TMEM52B both isoforms (Dharmacon, Lafayette, CO; 5′-GGGTACATCTCTGGTATATAT-3′), TMEM52B isoform 2 (5′-GGACGAGAUGUGAGGAAAATT-3′) or EGFR (5′-CGCAAAGUGUGUAACGGAAUATT-3′) using Lipofectamine 2000 for 48 h prior to analysis.

TMEM52B-specific siRNA (5′-GGGTACATCTCTGGTATATAT-3′) and scrambled siRNA (5′-ACTATATCTAGTTAGGTGCTG-3′) were subcloned into the pLKO.1 lentiviral shRNA vector (Addgene, Cambridge, MA, USA) digested with AgeI/EcoRI to generate pLKO.1-shTMEM52B and pLKO.1-shControl, respectively, according to the manufacturer’s instructions. TMEM52B-specific siRNA was also introduced into BglII/HindIII-digested pSUPER vector (OligoEngine, Seattle, WA). The EGFR-expressing vector was provided by Dr. M.-C. Hung [11]. Human E-cadherin-expressing vector was previously described [12]. Cells were transfected using Lipofectamine 2000 or electroporation (Invitrogen). At 48 h after transfection, cells were lysed for immunoblot analysis or harvested for further analysis. A cDNA fragment encoding the human E-cadherin extracellular domain was subcloned into the pCMV-Fc-myc vector [13]. The resulting recombinant soluble E-cadherin-Fc (sE-cad-Fc) fusion protein expression plasmid was transfected into HEK293E cells. At 48 h after transfection, the medium was changed to serum-free medium. Conditioned medium was collected and then subjected to affinity chromatography on a Protein A excellose column (Bioprogen, Daejon, Korea) to obtain purified sE-cad-Fc fusion protein.

### Generation of stable cell lines

To generate lentiviruses, pLKO.1-shTMEM52B or pLKO.1-shControl was co-transfected with Lentiviral Packaging Mix (Sigma, St Louis, MO) into Lenti-X-293 T cells (Clontech, Mountain View, CA) using Lipofectamine 2000, and virus-containing supernatants were harvested and concentrated at 48 h post-transfection. HCT-15 cells were transduced with the lentiviruses for 9 h in the presence of polybrene (5 μg/ml) and were subsequently selected with puromycin (15 μg/ml) for a week to establish stable clones.

### Immunoblot analysis

Whole-cell lysates were prepared using RIPA buffer as described previously [14] and analyzed using the following primary antibodies: anti-Histone H3, anti-β-actin and anti-GAPDH (Santa Cruz Biotechnology, Santa Cruz, CA); anti-myc (Upstate Biotechnology, Lake Placid, NY); anti-α-catenin, anti-β-catenin, anti-E-cadherin, and anti-integrin α5 (BD Biosciences, San Jose, CA); anti-α smooth muscle actin (αSMA) and anti-vimentin (Sigma); anti-phospho-c-Jun N-terminal kinase (JNK) (T183/Y185), anti-phospho-extracellular signal-regulated kinase 1/2 (ERK1/2), anti-ERK1/2, anti-phospho-AKT (S473), anti-AKT, anti-cyclin D1, anti-JNK, anti-phospho-c-Jun(S63), anit-phospho-ATF-2(T71), anti-c-Jun, anti-ATF-2, anti-Slug, anti-bcl-2, anti-survivin, anti-cleaved caspase-3, anti-PARP, anti-phospho-EGFR(Y1068), anti-EGFR, anti-phospho-β-catenin(S33, 37/T41), anti-phospho-β-catenin(S675), and anti-phospho-β-catenin(S552) (Cell Signaling, Danvers, MA); and anti-E-cadherin (R&D Systems, Minneapolis, MN).

To generate anti-TMEM52B antibodies, rabbits were immunized with a peptide (KQLPPTEKESTRIVDSWN), which was derived from the common region of both TMEM52B isoforms (amino acid residues from 166 to 183 of isoform 2), conjugated with KLH. After boostings, sera were collected to analyze anti-TMEM52B immunoactivity by ELISA (A-frontier, Seoul, Korea) and immunoblotting using cell lysates. The sera were further purified by affinity chromatography using protein A/G-agarose (Millipore, Billerica, MA).

### Subcellular fractionation

Subcellular fractions were prepared using the Compartmental Protein Extraction Kit (Millipore) according to the manufacturer’s instructions. The cytosolic fraction, nuclear fraction, plasma membrane-enriched fraction, and cytoskeletal fraction were analyzed by immunoblotting.

### Co-immunoprecipitation

HEK293E cells were transfected with pcDNA3.1-myc-TMEM52B and a vector expressing E-cadherin using Lipofectamine 2000. Two days after transfection, cells were lysed with co-immunoprecipitation buffer (10 mM Tris pH 7.4, 150 mM NaCl, 0.56 mM EGTA, 1% Triton X-100, 0.5% NP-40) supplemented with protease inhibitor (Complete, Roche). Lysates were centrifuged for 20 min at 10000 g and the resulting supernatant was precleared by incubation with protein A/G–agarose for 2 h at 4 °C. The precleared supernatant was immunoprecipitated using anti-myc at 4 °C for 16 h. The protein complexes were collected by incubation with protein A/G–agarose for 2 h at 4 °C and then washed four times with co-immunoprecipitation buffer. The protein complexes were eluted by boiling in sodium dodecyl sulfate sample buffer and analyzed by immunoblotting with anti-myc and anti-E-cadherin.

### Invasion assay

Invasion assays were performed as described previously [15]. Cells were plated in serum-free medium on Transwell inserts (Corning, NY) coated with 25 μg of Matrigel. The underside of the insert was pre-coated with 2 μg of collagen type I (Sigma). After incubation for 48 h at 37 °C in 5% CO_2_, inserts were fixed with 10% formalin and stained with 2% crystal violet. The number of cells that had invaded was counted in five representative (**×** 100) fields per insert or invasiveness was determined by calculating stained area relative to the total area using ImageJ software.

### Immunofluorescence staining

Cells were plated on coverslips and were transfected with shRNA for 48 h. Cells were then fixed for 15 min in 10% formalin and permeabilized for 5 min with 0.3% Triton X-100. After blocking in 10% normal goat serum, cells were stained using an anti-E-cadherin antibody (R&D Systems; 1:50) followed by an Alexa546-conjugated secondary antibody. EGF-treated transfected cells were fixed/permeabilized for 10 min in methanol. After blocking in 1% BSA, the cells were incubated with an anti-EEA1 (Cell Signaling; 1:100) or anti-LAMP2 antibody (Abcam, Cambridge, UK; 1:100) followed by a FITC-conjugated secondary antibody and then with an Alexa555-conjugated anti-EGFR antibody (Cell Signaling; 1:100) Cells were counterstained with 4,6-diamidino-2-phenylindole (DAPI; Sigma) to visualize nuclei. Mounted samples were visualized with a confocal microscope (LSM 510 META; Carl Zeiss, Jena, Germany). Intensity of E-cadherin staining was quantitated by ImageJ software. Quantitative co-localization analysis of EGFR and EEA1 or LAMP2 was performed by calculating Pearson’s correlation coefficient (r) using ImageJ software.

### Promoter reporter assay

For transfection, cells were seeded into 6-well plates at a density of 2 × 10^5^ cells/well and incubated for 24 h. Cells were then transfected with 2 μg of reporter plasmids DNA and 1.8 μg of the TMEM52B expression vector using Lipofectamine 2000. At 48 h post-transfection, firefly luciferase activity was measured using a Dual-luciferase reporter assay system (Promega, Southampton, UK). The transfection efficiency was normalized by measuring Renilla luciferase activity, encoded by the co-transfected Renilla luciferase vector (pRL-TK). The AP-1 cis-element reporter plasmids were purchased from Stratagene (La Jolla, CA). β-Catenin-responsive firefly luciferase reporter plasmid TOPFlash and the negative control FOPFlash were purchased from Millipore. The E-cadherin promoter (− 308/+ 41) reporter plasmid was previously described [15].

### Anchorage-independent soft agar assay

Cells were seeded at a density of 1 × 10^3^ cells/well in 6-well tissue culture plates in 0.4% agar (Sigma) over a 0.6% agar feed layer. Cells were allowed to grow at 37 °C in 5% CO_2_ for 13 days, and the number of resulting colonies was counted per well.

### Analysis of cell survival and proliferation

Cell survival under suspension culture conditions was determined. Briefly, cells were seeded into 96-well plates with an Ultra-Low Attachment Surface (Corning) at a density of 1.5–2 × 10^4^ cells/well and incubated for 3 or 4 days. Cells were then incubated with WST reagent (Ez-Cytox; Dogenbio, Seoul, Korea; one-tenth of the medium volume) and the amount of formazan dye formed was determined by measuring absorbance at 450 nm using a spectrophotometric microplate reader (BMG LABTECH GmbH, Ortenber, Germany).

Cells were seeded into 96-well plates at a density of 3 × 10^3^ cells/well and incubated for 48 h. Cell proliferation was determined by the colorimetric WST assay as described above. Proliferation rates were also estimated by the growth rate quotient (GRQ) as calculated by the equation: GRQ = (*N* –*N*_0_)/*t* × 1/ *N*_0_, where *N* is the final cell number, *N*_0_ is the initial cell number and t is the time elapsed between the two counting [16].

### Flow cytometry

To analyze TMEM52B localization at the cell surface, HEK293E cells were transfected with expression vectors for TMEM52B with a myc tag at the N-terminus for 48 h and then stained with PE-conjugated anti-myc antibody (Cell Signaling). Viable propidium iodide-negative cells were analyzed by flow cytometry.

To analyze internalization of EGFR in response to EGF treatment, cells were treated with EGF for 5–30 min at 37 °C to allow internalization or maintained at 4 °C. Cells were transferred to ice-cold buffer to stop the occurrence of internalization and stained with FITC-conjugated anti-EGFR antibody (Santa Cruz Biotechnology). Propidium iodide-negative cells were analyzed by flow cytometry to analyze residual EGFR on cell surface.

### Mouse xenograft model and circulating tumor cell (CTC) survival analysis

All animal procedures were performed in accordance with the guidelines of the Animal Care Committee at the Korea Research Institute of Bioscience and Biotechnology (KRIBB) and with approval of the bioethics committee of the KRIBB (KRIBB-AEC-19098). Nude mice (BALB/c-nude, 5-week-old females) were obtained from Nara Biotech (Seoul, Korea). HCT-15 stable cells (TMEM52B-suppressed cells and scrambled shRNA-transfected control cells) mixed with Matrigel (1:1) were injected subcutaneously into the right flank of each mouse (5 × 10^6^ cells per mouse; *n* = 4 per group). Body weight and tumor volume were measured twice per week. On day 44, mice were sacrificed, and dissected tumor masses were fixed in 10% formalin. The tumor volumes were calculated as follows: tumor volume = (**a** × **b**^2^) × 1/2, where **a** was the width at the widest point of the tumor and **b** was the maximal width perpendicular to **a**.

For CTC survival analysis (early metastasis model), HCT-15 stable cells (TMEM52B-suppressed cells and control cells) were injected via the tail vein into nude mice (5 × 10^6^ cells per mouse; *n* = 5 per group). Mice were sacrificed 24 h after injection and total DNA was isolated from the lungs as previously reported [17, 18]. To quantify survival and early seeding (arrest) of CTCs, human tumor cell contents present in mouse lungs were determined as previously reported [18, 19]. Briefly, real-time qPCR analysis of human prostaglandin E receptor 2 (PTGER2) genomic DNA was performed with a PTGER2-specific primer pair (5′-TACCTGCAGCTGTACGCCAC-3′ and 5′-GCCAGGAGAATGAGGTGGTC-3′) and a human PTGER2-specific probe (FAM 5′-TGCTGCTTCTCATTGTCTCG-3′ TAMRA) using QuantiTect Probe PCR Master Mix (Qiagen, Hilden, Germany) on a Rotor gene Q instrument (Qiagen) according to the manufacturer’s instructions. PCR was performed in triplicate with a final volume of 50 μl per reaction using 1 μg of total genomic DNA as a template. After denaturation for 15 min at 95 °C, the reaction was continued for 60 cycles of 94 °C for 15 s and 60 °C for 60 s. In parallel, a standard curve was generated using genomic DNA extracted from HCT-15 cells and nude mouse lungs. Standard curve samples included serial dilutions of mouse-only, human-only, or human plus mouse mixed samples of known DNA concentrations. A standard curve with the equation of the linear trend line was developed by plotting the mean Threshold Cycle (*Ct*) values on the y-axis versus the log amount of human genomic DNA on the x-axis.

### Immunohistochemistry and TUNEL assay

Formalin-fixed and paraffin-embedded 6 μm-thick tissue sections from xenograft tumors were processed for immunohistochemistry analysis as per the standard protocol. Sections were stained with anti-Ki67 antibody (SP6; Abcam) using the peroxidase technique. The proliferation index (%) was determined by calculating the number of Ki67-positive cells relative to the total number of cells, which consisted of at least 1000 cells per field. Serial sections were stained with anti-E-cadherin (1:100 dilution; R&D Systems) and anti-vimentin (1:100 dilution; Sigma) and the immune complexes were detected using the Vectastain Elite ABC-HRP kit (Vector Laboratories, Burlingame, CA). The sections were counterstained with hematoxylin.

TUNEL staining was performed to measure apoptosis in tumor tissue sections using In Situ Apoptosis Detection Kit (Abcam) according to the manufacturer’s instructions. The apoptosis index (%) was determined by calculating the number of TUNEL-positive cells relative to the total number of cells, which consisted of at least 1000 cells per field.

### Analysis of The Cancer Genome Atlas (TCGA) and kmPlotter data

cBioPortal (www.cbioportal.org) [20, 21] was used to analyze Cancer Cell Line Encyclopedia (CCLE; Broad, 2019) [22] and TCGA-generated human kidney renal clear cell carcinoma data (TCGA, Firehose Legacy). All patient samples where the mRNA expression profiles were available were included in our analysis per cancer study. Calculation of Pearson’s correlation coefficient (r) and survival curve analysis were performed using the cBioPortal webpage tools. Survival of lung [23], breast [24], liver [25], ovarian [26], rectal, and thyroid [27] cancer patients within previously published data sets was analyzed using KM-plotter (http://kmplot.com).

### Statistical analysis

Statistical analyses were performed using the Student’s *t*-test, one-way ANOVA (with GraphPad Prism 8 (GraphPad Software, CA)), Logrank test (for survival analysis), and Pearson’s test (for correlation analysis). *P* < 0.05 was considered statistically significant.

## Results

### Expression of TMEM52B in cell lines

**Fig. 1:**
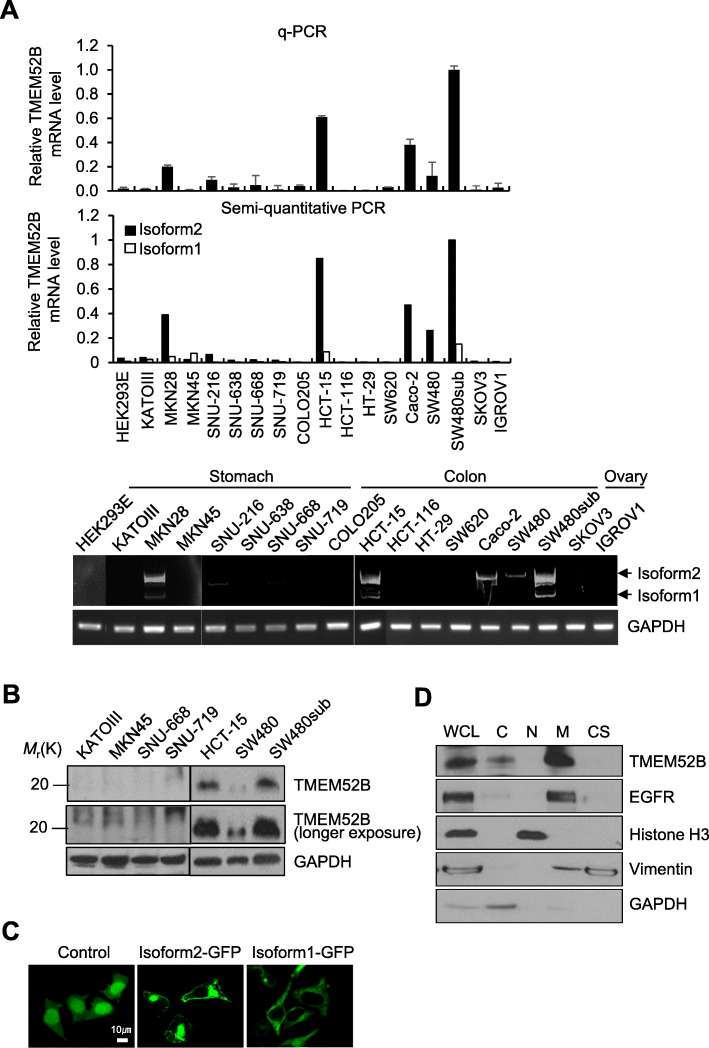
TMEM52B expression in cell lines. (A) Top, real-time quantitative PCR analysis of TMEM52B in various human cancer cell lines. β-actin was used as an internal control for input RNA. Middle, semi-quantitative RT-PCR analysis of TMEM52B. Densitometric quantification was performed on gel images. Bottom, gel images of semi-quantitative RT-PCR. All determinations were performed in three independent experiments. Values represent mean ± standard deviation (SD). *P < 0.05. (B) Immunoblot analysis of TMEM52B in human cancer cells. GAPDH was used as an internal control. (C) HEK293E cells were transfected with a GFP-fused TMEM52B-expression vector for 48 h before visualizing GFP. Scale bar, 10 μm. (D) Immunoblot analysis of subcellular localization of TMEM52B in SW480sub cells. WCL, whole-cell lysates; C, cytosolic fraction; N, nuclear fraction; M, plasma membrane-enriched fraction; CS, cytoskeletal fraction. GAPDH, Histone H3, EGFR, and vimentin were used as internal controls for the cytosolic, nuclear, and plasma membrane, and cytoskeletal fractions, respectively

To explore the expression of TMEM52B in various cancer cell lines, we performed RT-qPCR analysis. TMEM52B mRNA was highly expressed in SW480sub (a subclone of SW480, displaying elevated cell-matrix adhesion capacity), HCT-15, and Caco-2 colon cancer cells and was moderately expressed in MKN28 (gastric cancer), SW480 (colon cancer) cells. The mRNA was little to no detected in KATOIII, MKN45, SNU-216, SNU-638, SNU-668, SNU-719 (gastric cancer), Colo205, HCT-116, HT-29, SW620 (colon cancer), SKOV3, IGROV1 (ovarian cancer), and HEK293E cells (Fig. 1a, top panel). Notably, semi-quantitative PCR analyses, using primers that discriminate between TMEM52B isoforms, detected isoform 2 mRNA expression in SW480sub, HCT-15, Caco-2, SW480, and MKN28 cells. Isoform 1 mRNA expression was little or no detected (Fig. 1a middle and bottom panels), suggesting that isoform 2 was the major transcript. Immunoblot analysis using an antibody raised against a TMEM52B peptide (KQLPPTEKESTRIVDSWN) showed that the presumptive isoform 2 was substantially expressed in SW480ub and HCT-15 cells, and moderately expressed in SW480 cells, but not in KATOIII, MKN45, SNU-668, and SNU-719 cells (Fig. 1b).

To explore TMEM52B subcellular localization, HEK293E cells were transiently transfected with GFP-fused TMEM52B-expressing vectors. Substantive green fluorescence was detected at the cell membrane edges after isoform 2 transfections. Isoform 1 transfections resulted in diffused fluorescence in the cytoplasm and cell membranes (Fig. 1c). Neither isoform was present in the cell nuclei. In addition, HEK293E cells were transiently transfected with expression vectors for TMEM52B with a myc tag at the N-terminus. Flow cytometry analysis showed that an anti-myc antibody bound to HEK293E cells transfected with isoform 2 and isoform 1 (Supplementary Fig. S1A and B), confirming that TMEM52B localized to the plasma membrane. Notably, this antibody bound more to HEK293E cells transfected with isoform 2 than to HEK293E cells transfected with isoform 1. Subcellular fractionation and immunoblot analyses showed a strong signal intensity in the plasma membrane-enriched fraction, and a moderate signal intensity in the cytoplasm fraction from SW480sub cells (Fig. 1d). These results indicate that TMEM52B expression was mainly present on the plasma membrane.

### Suppression of TMEM52B promoted cancer cell invasion and survival through activation of MAPKs and AKT signaling pathways

**Fig. 2:**
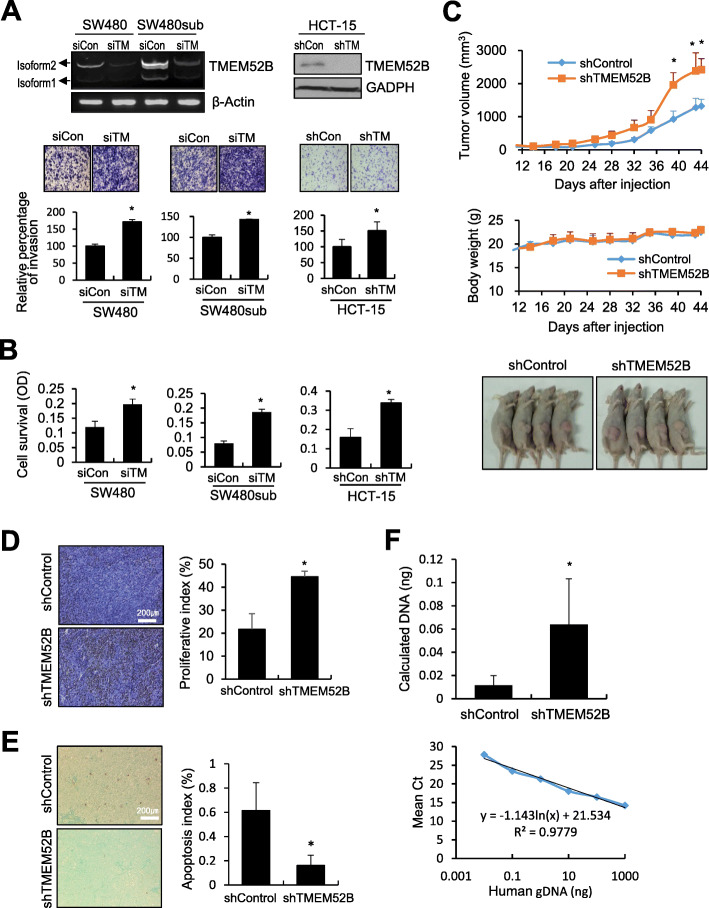
Suppression of TMEM52B promotes cancer cell invasion and survival in vitro, and tumor growth and early metastasis in vivo. (A, B) Cells were transfected with siRNA or shRNA specific to TMEM52B for 48 h. (A) Transfected cells (2 × 10^4^ cells/well) were allowed to invade Matrigel on Transwell inserts for 48 h. The number of cells that had invaded was counted in five representative (100×) fields per Transwell insert. Knockdown of TMEM52B was analyzed by semi-quantitative RT-PCR or by immunoblot. (B) To induce anoikis, transfected cells were seeded into 96-well plates with an Ultra-Low Attachment surface and grown for up to 3 days. Cell viability was determined by the colorimetric WST assay. (C) In vivo tumor growth analysis. Stable TMEM52B-suppressed HCT-15 cells (5 × 10^6^ cells/mouse) were injected subcutaneously into the flanks of nude mice (*n* = 4) as described in Materials and Methods. Tumor volume and body weight were measured for 44 days. Tumor volume was calculated using the formula, length × width^2^/2. Photos of tumor-bearing mice on day 44. (D, E) Ki67 (D) and TUNEL (E) staining of tumor sections from (C) was performed to measure the level of cell proliferation and apoptosis, respectively. Representative images are shown. The proliferative index (%) or apoptosis index (%) was determined by calculating the number of Ki67 or TUNEL-positive cells relative to the total number of cells (at least 1000 cells per field). Five randomly selected fields from tumor sections per mouse were analyzed. Scale bars, 200 μm. (F) Stable HCT-15 cells (5 × 10^6^ cells/mouse) were intravenously injected into nude mice (*n* = 5) as described in Materials and Methods. At 24 h after injection, the lungs were removed to extract total DNA. Real-time qPCR analysis was performed for human PTGER2 using total DNA extracted from the lungs. The amount of human genomic DNA initially present in a qPCR reaction tube was estimated (top) from the standard curve generated by qPCR using human total DNA extracted from HCT-15 parental cells, which was spiked into mouse total DNA from lungs of nude mice (bottom). All in vitro determinations were performed in three independent experiments. Values represent mean ± SD. *P < 0.05. siCon, scrambled control siRNA, shCon, scrambled control shRNA; siTM, TMEM52B-specific siRNA; shTM, TMEM52B-specific shRNA

To explore the effect of suppressed TMEM52B expression on cell growth, invasion, and survival, SW480, SW480sub, and HCT-15 cells were transiently transfected with siRNA or shRNA specific to both isoform 1 and isoform 2. TMEM52B suppression significantly increased invasion of SW480, SW480sub, and HCT-15 cells by 71, 43, and 51%, respectively (Fig. 2a). Cell survival under suspension conditions was also increased by 67, 138, and 113%, following TMEM52B suppression in SW480, SW480sub, and HCT-15 cells, respectively (Fig. 2b). On the other hand, cell proliferation was not substantially affected by TMEM52B depletion (Supplementary Fig. S1C and D), whereas anchorage-independent growth of cells was significantly enhanced by TMEM52B suppression (Supplementary Fig. S2A and B).

To evaluate the effect of TMEM52B suppression in vivo, stable HCT-15 cells with suppressed TMEM52B expression were generated and characterized (Supplementary Fig. S2A–D); consistent with Fig. 2a and b, TMEM52B suppression enhanced cell survival and invasion, which was accompanied by enhanced EGFR phosphorylation and reduced E-cadherin (see below). These cells were injected subcutaneously into the flanks of nude mice. Tumor growth was significantly increased in mouse xenografts with TMEM52B-suppressed cells compared to those with scrambled shRNA-transfected control cells (Fig. 2c). Immunostaining of tumor sections from mice injected with TMEM52B-suppressed cells showed higher levels of proliferative Ki67-positive cells compared with the control-treated mouse tumors (Fig. 2d). Additionally, TUNEL analysis (Fig. 2e) of these same tumor sections displayed significantly lower levels of apoptosis compared with that of the tumors from mice injected with control cells. E-cadherin was substantially detected and vimentin was not apparently detected in tumor sections of mice injected with TMEM52B-expressing control cells, while vimentin but not E-cadherin was moderately detected in tumor sections of mice injected with TMEM52B-suppressed cells (Supplementary Fig. S2E). We also examined the effect of TMEM52B suppression on the survival of, and subsequent seeding/arrest of, circulating tumor cells (CTCs) in vivo. As an early metastasis model, TMEM52B-suppressed stable HCT-15 cells were intravenously injected into nude mice. At 24 h after injection, mice were sacrificed, and the lungs immediately removed for total DNA extraction to determine tumor cell content. Real-time qPCR analysis revealed that the amount of human genomic DNA was significantly higher in the lungs of mice injected with TMEM52B-suppressed cells than in the control-treated mouse lungs (Fig. 2e). This indicates that TMEM52B suppression promotes CTC survival to support early metastasis.

**Fig. 3:**
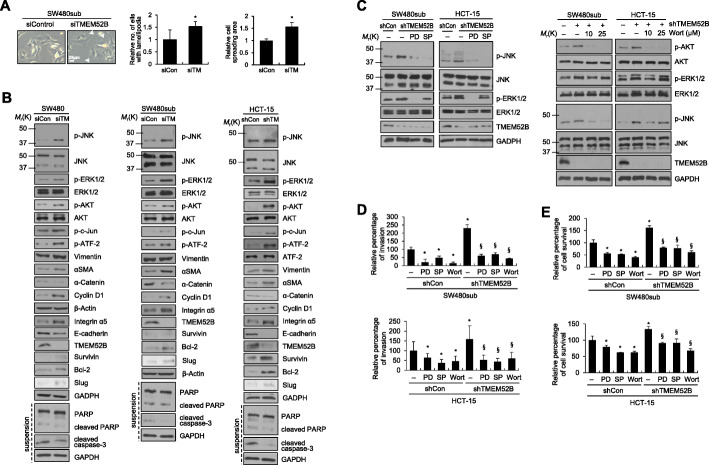
MAPK and AKT signaling pathways are required for cancer cell invasion and survival mediated by TMEM52B suppression. (A) Phase-contrast images of SW480sub control cells and TMEM52B-knockdown cells. Arrowheads indicate representative membrane ruffling or protrusive spot. Number of cells displaying lamellipodia or protrusive spots (more than 3) were counted. Cell spreading area per cell (at least 200 cells) was calculated using ImageJ software. Values represent mean ± SD. *P < 0.05. Scale bar, 25 μm. (B) Cells were transfected with siRNA or shRNA specific to TMEM52B for 48 h prior to lysis for immunoblot analysis or RT-PCR analysis. Transfected cells were incubated for 48 h under suspension culture conditions prior to lysis for immunoblot analysis of cleaved caspase-3 and PARP. (C) Cells were transfected with shRNA specific to TMEM52B for 24 h and then treated with pharmacological inhibitors: PD098059 (5 μM SW480sub and 20 μM HCT-15); SP600125, 15 μM; wortmannin, 10 μM or 25 μM, or 0.1% DMSO as a vehicle control for 24 h before whole-cell lysates were prepared for immunoblotting. (D, E) Cells were transfected with shRNA specific to TMEM52B for 48 h. Invasion (D) and cell survival (E) assays were performed as described in Fig. 2A and B in the presence of pharmacological inhibitors: PD098059 (5 μM SW480sub and 20 μM HCT-15); SP600125, 15 μM; wortmannin, 10 μM, or DMSO. Cell invasion was determined by calculating the cell-stained area relative to the total area using ImageJ software. All determinations were performed in three independent experiments. Values represent mean ± SD. *P < 0.05 compared with shControl + DMSO; ^§^*P* < 0.05 compared with shTMEM52B + DMSO

Comparison of the cell morphology revealed enhanced spreading and formation of membrane ruffle and protrusive spots in TMEM52B-suppressed SW480sub cells (Fig. 3a). Immunoblot analyses showed that TMEM52B suppression induced phosphorylation of JNK (moderately in HCT-15 cells), ERK1/2, and AKT in SW480, SW480sub, and HCT-15 cells. The phosphorylation of c-Jun and ATF-2, and the expression of cyclin D1, were also enhanced by TMEM52B knockdown. In addition, TMEM52B suppression upregulated the mesenchymal markers, including vimentin (in SW480 and HCT-15 cells), α smooth muscle actin, integrin α5, and Slug (moderately). This suppression also downregulated epithelial markers, including E-cadherin (in SW480 and HCT-15 cells; E-cadherin was not apparently detected in SW480sub cells) and α-catenin (Fig. 3b). TMEM52B knockdown enhanced expression of the anti-apoptotic factors bcl-2 and survivin (in HCT-15 cells). TMEM52B suppression reduced the levels of cleaved caspase-3 and PARP (in SW480 and HCT-15 cells) under suspension culture conditions (Fig. 3b). These results indicate that TMEM52B suppression promotes EMT events, leading to cancer cell invasion and survival. Using a reporter assay, TMEM52B suppression significantly activated an AP-1 cis-element reporter plasmid (AP-1 reporter), in SW480 and HCT-15 cells (Supplementary Fig. S3A), indicating that TMEM52B suppression increases AP-1 transcriptional activity.

To determine the role of JNK, ERK1/2, and AKT signaling in TMEM52B suppression-mediated cancer cell survival and invasion, SW480sub and HCT-15 cells were transiently transfected with TMEM52B-specific shRNA for 24 h and then treated with the following pharmacological inhibitors for 24 h before analysis: dimethyl sulfoxide (vehicle), PD098059 (a specific MEK/ERK inhibitor), SP600125 (a specific JNK inhibitor), or wortmannin (a specific PI3K/AKT inhibitor). Immunoblot analysis showed signaling suppression by these inhibitors. Inhibition of MEK/ERK also moderately reduced JNK phosphorylation in both cells, implying that ERK may be involved in JNK activation. Inhibition of PI3K/AKT also reduced JNK phosphorylation in SW480sub cells, implying that PI3K/AKT may be involved in JNK activation (Fig. 3c). Inhibition of MEK/ERK, JNK, and PI3K/AKT significantly suppressed both basal and TMEM52B suppression-induced cell invasion (Fig. 3d) and survival (Fig. 3e).

In addition, siRNA suppression of only isoform 2 (the major isoform) promoted SW480sub and HCT-15 cell invasion and survival to an extent similar to that seen after suppression of both isoforms (Supplementary Fig. S3B), confirming that isoform 2 is the major isoform and that cellular functions mediated by TMEM52B (suppression) are largely attributed to isoform 2.

Together, these results suggest that TMEM52B suppression promotes cancer cell invasion and survival through activation of ERK1/2, JNK, and AKT signaling pathways.

### TMEM52B suppression promoted invasion and cell survival in an EGFR-dependent manner

**Fig. 4:**
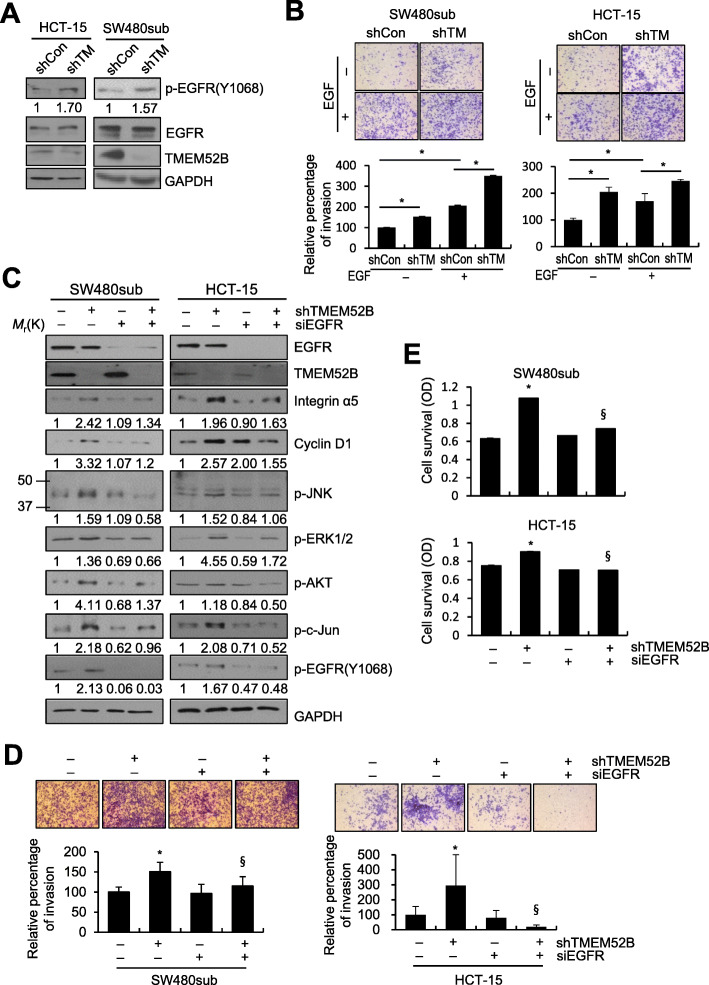
TMEM52B suppression promotes invasion and cell survival in an EGFR-dependent manner. (A) Immunoblot analysis of EGFR phosphorylation in cells transfected with shRNA specific to TMEM52B. (B) Cells were transfected with shRNA specific to TMEM52B for 48 h. Transfected cells were allowed to invade Matrigel in the absence or presence of EGF (10 ng/ml) for 48 h. Cell invasion was determined by calculating the cell-stained area relative to the total area using ImageJ software. (C-E) Cells were co-transfected with siRNA specific to EGFR, and shRNA specific to TMEM52B for 48. (C) Transfected cells were lysed for immunoblot analysis. Densitometric quantification was performed on the immunoblots using GAPDH as a loading control. Transfected cells were subjected to invasion (D) and cell survival (E) assays as described in Fig. 2A and B. All determinations were performed in three independent experiments. Values represent mean ± SD. *P < 0.05 compared with shControl + siControl; ^§^*P* < 0.05 compared with shTMEM52B + siControl

Next, we explored the upstream signaling responsible for TMEM52B suppression-mediated signaling pathway activation and invasion. We hypothesized that TMEM52B may activate several signaling pathways by modulating specific receptor tyrosine kinase activity due to TMEM52B being localized to the cell surface and not having any known enzymatic domain. Among the screened molecules, EGFR phosphorylation at Tyr1068 increased in TMEM52B-suppressed HCT-15 and SW480sub cells (Fig. 4a). As described below, exogenous EGF-induced phosphorylation of EGFR was also enhanced by TMEM52B suppression. Both basal cell invasion, and that induced by exogenous EGF, were enhanced by TMEM52B suppression (Fig. 4b).

To determine whether EGFR was required for TMEM52B suppression-mediated signaling events and invasion, SW480sub and HCT-15 cells were transiently co-transfected with EGFR-specific siRNA and a TMEM52B-specific shRNA vector. Immunoblot analysis demonstrated that the TMEM52B suppression-mediated phosphorylation of JNK, ERK1/2, c-Jun, and AKT was reversed following the suppression of EGFR expression. EGFR suppression also reduced TMEM52B suppression-mediated expression of both integrin α5 and cyclin D1 (Fig. 4c). TMEM52B suppression-induced invasion (Fig. 4d) and cell survival (Fig. 4e) were both significantly reduced by EGFR knockdown. These results suggest that the suppression of TMEM52B mediated activation of intracellular signaling and promoted invasion and cell survival, in an EGFR-dependent manner.

### TMEM52B suppression promoted phosphorylation and internalization of EGFR and enhanced downstream signaling

**Fig. 5:**
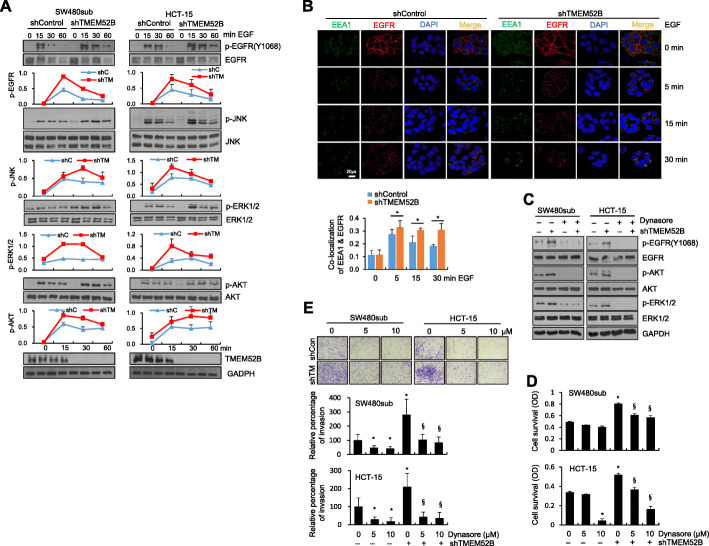
TMEM52B suppression promotes phosphorylation and internalization of EGFR and downstream signaling. (A) Cells were transfected with shRNA specific to TMEM52B for 48 h and treated with EGF (10 ng/ml) for the indicated time prior to lysis for immunoblot analysis. Densitometric quantification was performed on the immunoblots; phosphorylated proteins were normalized against the corresponding total protein. (B) SW480sub cells were transfected with shRNA for 48 h and treated with EGF (100 ng/ml) for the indicated times. EGFR, EEA1, and DAPI were visualized; green for EEA1, red for EGFR, and blue for DAPI staining. Scale bar, 20 μm. Co-localization of EGFR and EEA1 was quantitated by calculating Pearson’s correlation coefficient using ImageJ software. Values represent mean ± SD. *P < 0.05. (C) Cells transfected with shRNA specific to TMEM52B for 42 h were treated with dynasore (160 μM) or DMSO as a vehicle control for 6 h and then treated with EGF (10 ng/ml) for 5 min prior to lysis for immunoblotting. (D, E) Cells were transfected with shRNA specific to TMEM52B for 48 h. Cell survival (D) and invasion (E) assays were performed as described in Fig. 2A and B in the presence of dynasore (5 μM or 10 μM) and EGF (10 ng/ml). Cell invasion was determined by calculating the cell-stained area relative to the total area using ImageJ software. All determinations were performed in three independent experiments. Values represent mean ± SD. *P < 0.05 compared with shControl + DMSO; ^§^*P* < 0.05 compared with shTMEM52B + DMSO

We further explored the effect of TMEM52B suppression on EGFR in response to EGF. TMEM52B-suppressed SW480sub and HCT-15 cells responded to EGF treatment with enhanced and sustained activation of EGFR. These cells also exhibited activation of downstream signaling mediators ERK1/2, JNK, and AKT (Fig. 5a). Upon ligand binding, EGFR is involved in a series of trafficking events, which ultimately regulate its signal amplification, cell survival, and invasiveness. We therefore investigated the intracellular distribution of EGFR using immunofluorescence staining. In unstimulated SW480sub cells, EGFR was predominantly localized to cell surface membranes. Following EGF stimulation, internalized EGFR co-localized with EEA1-positive early endosomes in both TMEM52B-suppressed and control cells. TMEM52B-suppressed cells displayed sustained EGFR localization to early endosomes compared to control cells for up to 30 min (Fig. 5b). In addition, internalized EGFR that co-localized with LAMP2-positive lysosomes, was observed less in TMEM52B-suppressed cells than in control cells at 60 min (Supplementary Fig. S4A). Consistently, flow cytometry analysis showed that residual EGFR levels at the cell surface were lower in TMEM52B-suppressed SW480sub cells, following EGF stimulation, compared to EGFR levels in control cells at both 15 and 30 min (Supplementary Fig. S4B).

In order to examine the functional significance of internalized EGFR signaling (localized to early endosome), dynasore, a cell-permeable dynamin inhibitor, was used to block EGFR endocytosis. As expected, EGFR endocytosis was inhibited by dynasore in TMEM52B-suppressed cells (Supplementary Fig. S4C). Immunoblot analysis showed that EGF-induced activations of EGFR, AKT, and ERK1/2 were substantially reduced in TMEM52B-suppressed SW480sub and HCT-15 cells (Fig. 5c). These results indicate the functional significance of endosomal EGFR in promoting AKT and ERK1/2 downstream signaling. Consistent with the result of Fig. 5c, inhibition of EGFR endocytosis significantly reduced TMEM52B suppression-mediated cell survival (Fig. 5d) and invasion (Fig. 5e).

Together, our results indicate that TMEM52B suppression promotes the internalization and sustained accumulation of EGFR in the endosomal compartment in response to EGF treatment, resulting in enhanced activation of EGFR and its downstream signaling mediators AKT and ERK1/2 to drive cancer cell survival and invasion.

### TMEM52B overexpression suppressed invasion and survival of cancer cells and reduced phosphorylation of EGFR, MAPKs, and AKT

**Fig. 6:**
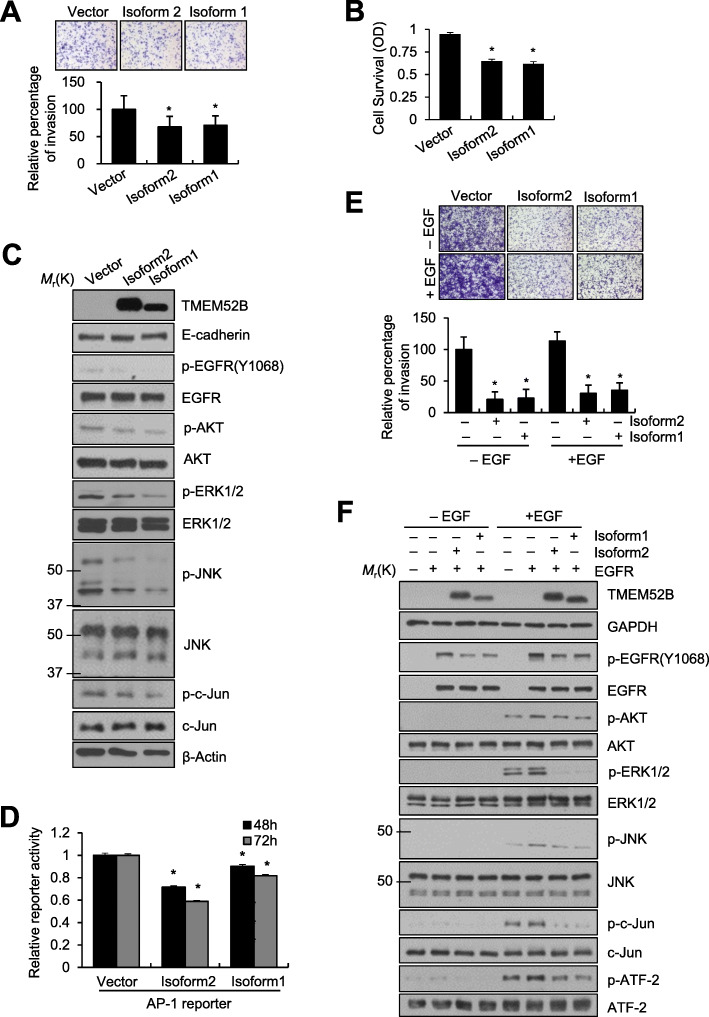
TMEM52B overexpression suppresses cancer cell invasion and survival and reduces phosphorylation of EGFR, MAPKs, and AKT. (A-C) SW480 cells were transfected with a TMEM52B-expression vector for 48 h. Invasion (A) and cell survival (B) assays were performed as described in Fig. 2A and B, respectively. (C) Transfected cells were lysed for immunoblot analysis. Anti-myc was used to detect myc-tagged TMEM52B. (D) SW480 cells were co-transfected with a TMEM52B-expression vector and an AP-1 reporter plasmid for 48 h. AP-1 activity was determined by a reporter assay as described in Materials and Methods. (E, F) HEK293E cells were co-transfected with an EGFR-expression vector and a TMEM52B-expression vector for 48 h. (E) Transfected cells (2 × 10^4^ cells) were allowed to invade Matrigel for 48 h in the absence or presence of EGF (10 ng/ml). Cell invasion was determined by calculating the cell-stained area relative to the total area using ImageJ software. All determinations were performed in three independent experiments. Values represent mean ± SD. *P < 0.05. (F) Transfected cells were lysed for immunoblot analysis. An anti-myc antibody was used to detect myc-tagged TMEM52B

We next explored whether TMEM52B overexpression suppressed cancer cell invasion and survival. SW480 cells were transiently transfected with TMEM52B-expressing vectors for 48 h prior to invasion and cell survival assays. Overexpression of both isoforms significantly reduced cancer cell invasion (Fig. 6a) and survival (Fig. 6b). Immunoblot analysis showed that overexpression of both isoforms reduced phosphorylation of EGFR, AKT, JNK, ERK1/2, and c-Jun (Fig. 6c). AP-1 transcriptional activity in SW480 cells was also decreased in response to expression of both isoforms (Fig. 6d). However, stable TMEM52B-overexpressing SW480 cells were not successfully established in our study, possibly due to reduced cell viability mediated by TMEM52B overexpression. In addition, enforced expression of TMEM52B isoforms in HCT-116 cells, which display little to no TMEM52B expression, reduced phosphorylation of EGFR, AKT, JNK, ERK1/2, and c-Jun along with reduced cancer cell invasion and survival (Supplementary Fig. S5), confirming the effects of the TMEM52B isoforms. On the other hand, TMEM52B overexpression did not substantially elevate E-cadherin expression in SW480 and HCT-116 cells (Fig. 6c and Supplementary Fig. S5C).

To examine the effect of TMEM52B overexpression on EGFR activity and EGFR-mediated cellular functions, HEK293E cells were co-transfected with TMEM52B- and EGFR-expressing vectors. The overexpression of both isoforms significantly suppressed both basal and EGF-induced cell invasion (Fig. 6e) (Notably, EGFR overexpression alone resulted in enhanced EGFR phosphorylation and increased invasiveness without the addition of exogenous EGF (data not shown). Therefore, the moderate effect observed after exogenous EGF treatment may have been due to a certain level of EGFR overexpression-mediated EGFR activation). Immunoblot analysis showed that EGFR overexpression enhanced phosphorylation of EGFR, AKT, ERK1/2, JNK, c-Jun (moderately), and ATF-2, and these changes were attenuated by both isoforms (Fig. 6f). These results suggest that TMEM52B reduces EGFR activation and that of downstream signaling pathways, leading to suppression of cancer cell invasion and survival.

### TMEM52B suppression reduced E-cadherin stability to enhance β-catenin activity and promoted the generation of soluble E-cadherin fragment that contributed to EGFR activation

**Fig. 7:**
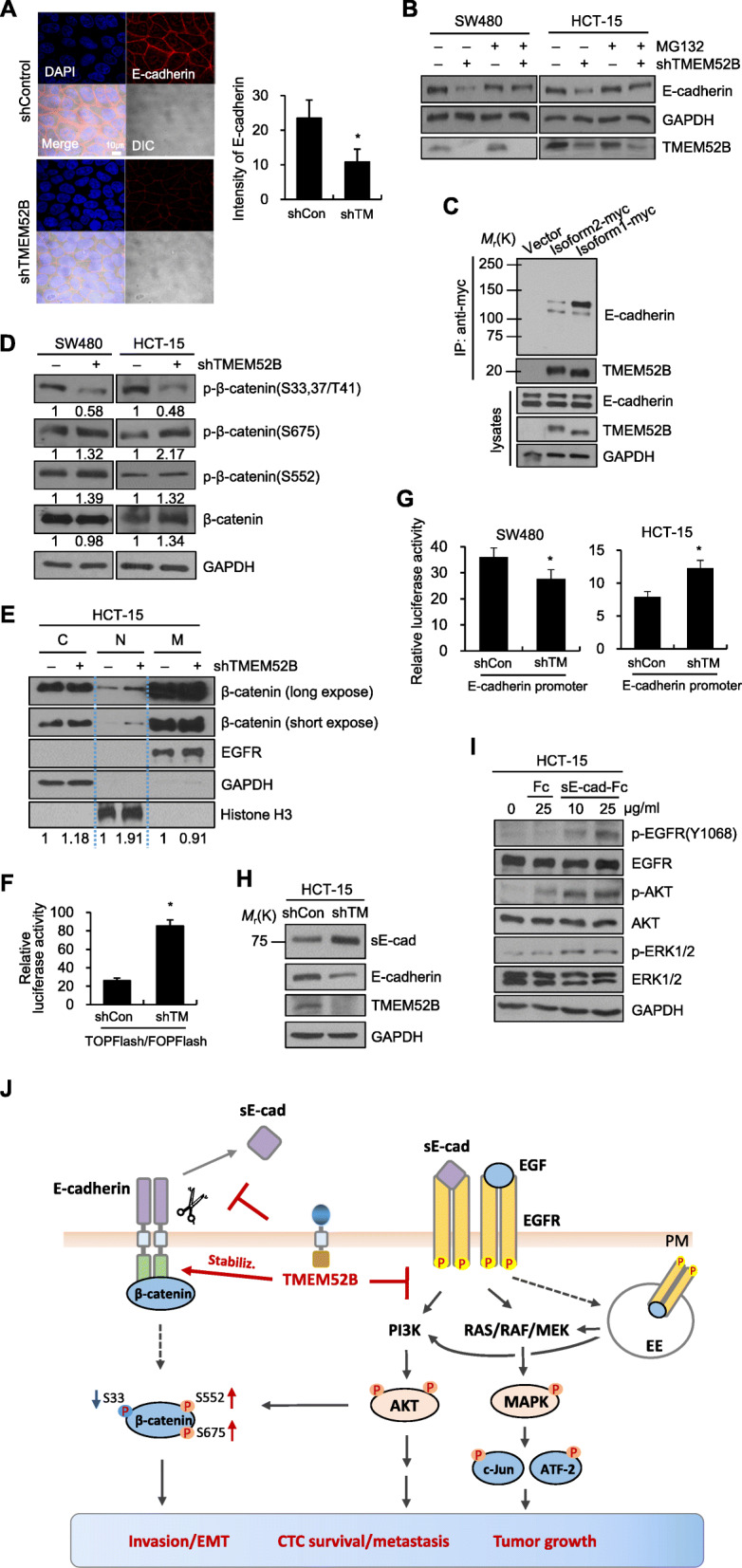
TMEM52B suppression reduces E-cadherin stability and promotes extracellular shedding of E-cadherin, resulting in EGFR phosphorylation. (A) HCT-15 cells transfected with shRNA specific to TMEM52B for 48 h were immunofluorescent stained for E-cadherin (red) and nuclei (blue). Differential interference contrast (DIC) images are also shown. Scale bar, 10 μm. The level of E-cadherin-staining was calculated based on the ratio of the E-cadherin-positive area to the total observation area using ImageJ software. At least 7 random fields were analyzed. (B) Cells transfected with shRNA specific to TMEM52B for 42 h were treated with MG132 (2 μM) for 6 h before lysis for immunoblot analysis. (C) Co-immunoprecipitation analysis of the interaction between TMEM52B and E-cadherin in HEK293E cells co-transfected with myc-tagged TMEM52B and E-cadherin. Whole-cell lysates were immunoprecipitated with anti-myc and analyzed by immunoblotting with anti-myc or anti-E-cadherin. (D, E) Cells were transfected with shRNA specific to TMEMT52B for 48 h. (D) Transfected cells were lysed for immunoblotting. (E) A cytosolic fraction (denoted by C), nuclear fraction (N), and plasma membrane-enriched fraction (M) were prepared from transfected HCT-15 cells for immunoblot analysis of β-catenin. GAPDH, histone H3, and EGFR were used as internal controls for the cytosolic, nuclear, and plasma membrane fractions, respectively. Densitometric quantification was performed on the immunoblots using GAPDH or a fractional internal control as a loading control. (F) Transcriptional activity of β-catenin was analyzed using the TOP/FOP reporter system. HCT-15 cells were co-transfected with shRNA specific to TMEM52B and reporter plasmid for 48 h. Firefly luciferase activity was normalized against the Renilla luciferase activity and fold increases in TOPFlash activity compared to FOPFlash activity were calculated. (G) Cells were co-transfected with shRNA specific to TMEM52B and an E-cadherin promoter (− 308/+ 41) reporter plasmid for 48 h. E-cadherin promoter reporter activity was then measured. All determinations were performed in three independent experiments. Values represent mean ± SD. *P < 0.05. (H) HCT-15 cells were transfected with TMEM52B-specific shRNA for 48 h and the conditioned media from transfected cells collected for an additional 48 h. Lysates (intact E-cadherin) from transfected cells, and conditioned media (soluble E-cadherin; denoted as sE-cad) from cells were analyzed by immunoblotting. (I) Cells were serum-starved for 24 h and then treated with recombinant extracellular E-cadherin protein fused with Fc for 8 h. Cells were also treated with 25 μg/ml recombinant Fc protein as a negative control. Lysates were prepared and analyzed by immunoblotting. (J) A schematic representation illustrating the pathways for TMEM52B action in human cancer cells. TMEM52B suppression promoted activation and internalization of EGFR with enhanced MAPK and AKT signaling activity, leading to enhanced cell survival and invasion. In addition, TMEM52B suppression reduced E-cadherin stability, likely due to a reduced association between it and E-cadherin, resulting in enhanced β-catenin transcriptional activity. TMEM52B suppression-mediated generation of a soluble E-cadherin fragment, at least partially, contributed to EGFR activation. EE, early endosome. Nucleus was not distinguished. Dashed arrows indicate translocation

We next explored the mechanism of reduced E-cadherin expression following TMEM52B suppression. Immunofluorescence analysis showed that TMEM52B-suppressed HCT-15 cells displayed significantly less E-cadherin, whereas scrambled siRNA-transfected cells maintained E-cadherin expression at organized cell-to-cell junctions (Fig. 7a). We examined whether the TMEM52B suppression modulated E-cadherin protein stability. SW480 and HCT-15 cells were transiently transfected with TMEM52B-specific shRNA for 42 h and then treated with MG132, a proteasome inhibitor, for 6 h before lysis. TMEM52B suppression reduced E-cadherin levels, and this effect was substantially reversed by MG132 treatment (Fig. 7b). Co-immunoprecipitation studies, using whole-cell lysates from HEK293 cells co-overexpressing myc-tagged TMEM52B and E-cadherin, revealed that both isoforms co-precipitated with E-cadherin (Fig. 7c) (Of note, two bands were detected by anti-E-cadherin, which may be due to E-cadherin modification such as phosphorylation in these cells). These results suggest that TMEM52B binds to E-cadherin and thus enhances its stability.

In addition, suppression of TMEM52B reduced β-catenin phosphorylation at Ser33, 37/Thr41, but enhanced phosphorylation at Ser675 (in SW480 and HCT-15 cells) and Ser552 (in SW480 cells) (Fig. 7d), resulting in enhanced nuclear transport of β-catenin (Fig. 7e). Those changes of β-catenin phosphorylation at Ser33, 37/Thr41, and Ser552 may be mediated directly or indirectly (through GSK-3) by AKT [28, 29]. Consistently, a reporter assay revealed that transcriptional activity of β-catenin was substantially increased by TMEM52B suppression in HCT-15 cells (Fig. 7f). On the other hand, TMEM52B suppression moderately reduced E-cadherin promoter activity in SW480 cells, but not in HCT-15 cells (Fig. 7g), suggesting that TMEM52B suppression may reduce E-cadherin expression at the transcriptional level in a cell- or context-dependent manner.

Recently, it has been reported that soluble E-cadherin, an extracellular proteolytic fragment of E-cadherin, is elevated in the urine or serum of cancer patients, and can activate EGFR family members [6]. Immunoblot analysis of conditioned medium revealed that an extracellular domain fragment of E-cadherin (~ 80 kDa) was shed from TMEM52B-suppressed HCT-15 cells (Fig. 7h), so we examined whether this fragment of E-cadherin may play a role in activation of EGFR. Immunoblot analysis showed that EGFR phosphorylation was enhanced in HCT-15 cells following treatment with purified recombinant E-cadherin fragment protein compared to mock protein or no treatment (Fig. 7i). The phosphorylation of ERK1/2 and AKT was also enhanced by treatment with soluble E-cadherin fragment (Fig. 7i).

Together, these results suggest that TMEM52B suppression reduces E-cadherin expression, potentially through modulation of E-cadherin stability, and promotes the extracellular shedding of E-cadherin, leading to increased EGFR phosphorylation.

### Clinical significance of TMEM52B in human cancers

**Fig. 8:**
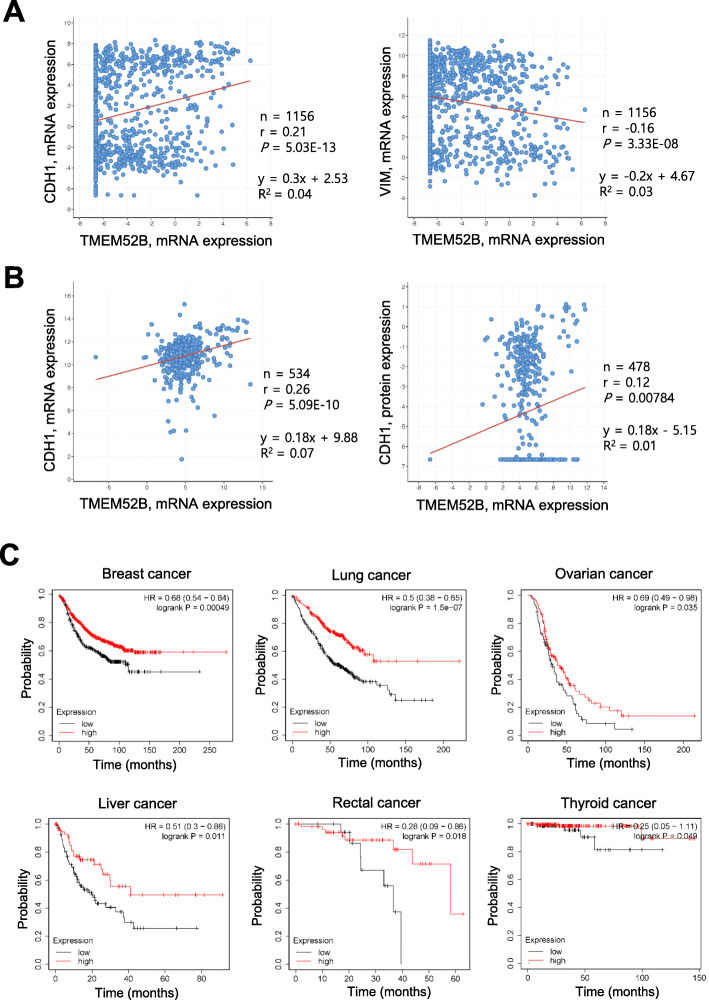
Clinical significance of TMEM52B in human cancers. (A) Scatter plots examining TMEM52B mRNA expression (x-axis) and E-cadherin (left) or vimentin (right) mRNA expression (y-axis) from CCLE data (Broad, 2019). (B) Scatter plots examining TMEM52B mRNA expression (x-axis) and E-cadherin mRNA (left) or E-cadherin protein (right) expression (y-axis) from kidney renal clear cell carcinoma data (TCGA, Firehose Legacy). Correlations were statistically analyzed using the Pearson test. Equations were automatically generated using the cBioPortal webpage tool. (C) TMEM52B expression was correlated with the survival of cancer patients with tumors expressing low E-cadherin levels (below the median) when analyzed using the KM-Plotter. Kaplan-Meier analysis showed the probability of relapse-free survival from breast (*n* = 882), overall survival from lung (*n* = 571), post-progression survival from ovarian (*n* = 191), relapse-free survival from liver (*n* = 158), overall survival from rectal (*n* = 82), and overall survival from thyroid (*n* = 251) cancer patients data in relation to TMEM52B mRNA expression. TMEM52B expression was stratified as high vs. low according to an auto-select best cutoff value, and survival plots based on previously published data sets were generated using http://kmplot.com. *P* values were calculated by the Logrank test

We examined whether TMEM52B expression correlated with E-cadherin expression in various cancer cell lines and in human cancers. Correlations were determined by calculating Pearson’s correlation coefficients (r). The analysis of various cancer cell lines from Cancer Cell Line Encyclopedia (CCLE) data demonstrated that TMEM52B mRNA expression was positively correlated with E-cadherin mRNA expression (*n* = 1156, r = 0.21, *P* = 5.03E− 13), whereas TMEM52B mRNA level was negatively correlated with vimentin mRNA (n = 1156, r = − 0.16, *P* = 3.33E− 08) (Fig. 8a). Analysis of kidney renal clear cell carcinoma data (TCGA, Firehose Legacy) showed that TMEM52B mRNA expression was positively correlated with E-cadherin mRNA expression (*n* = 534, r = 0.26, *P* = 5.09E− 10) and E-cadherin protein expression (*n* = 478 for protein data, r = 0.12, *P* = 0.00784) (Fig. 8b).

We also found that high TMEM52B expression in lung and breast cancer patients was significantly correlated with increased overall survival and relapse-free survival, respectively, when survival within previously published data sets was analyzed using the KM-plotter. TMEM52B expression in liver cancer patients showed a tendency for a positive correlation with increased relapse-free survival (Supplementary Fig. S6A-C). We also found that kidney cancer patients with tumors that highly expressed TMEM52B (Z > 1.5) had a significantly better overall survival rate than the remaining patients (Z ≤ 1.5) (Supplementary Fig. S6D) from the analysis of TCGA-generated kidney cancer data (TCGA, Firehose Legacy). Furthermore, high expression of TMEM52B in lung, breast, ovarian, liver, rectal, and thyroid cancer patients was significantly correlated with increased survival when only patients with tumors that expressed E-cadherin, at relatively low levels (below the median), were included in the KM-plotter analysis (Fig. 8c), suggesting that TMEM52B expression may compensate for relatively low levels of E-cadherin to suppress tumor progression, resulting in better survival.

## Discussion

We report here that TMEM52B plays a role in cancer cell invasion and survival, in an EGFR-dependent manner, to reduce tumor growth and early metastasis. TMEM52B suppression activated EGFR and downstream MAPKs and AKT signaling. In addition, TMEM52B suppression promoted EGFR internalization and sustained EGFR activation and signaling. TMEM52B enhanced E-cadherin protein stability through binding and thus blocking its proteasomal degradation and extracellular shedding. TMEM52B suppression-mediated generation of a soluble E-cadherin fragment, at least partially, contributed to EGFR activation. These results provide the first evidence that TMEM52B has tumor suppressor-like activity and is a novel modulator of EGFR and E-cadherin (Fig. 7j). The clinical data analysis showed that TMEM52B expression was positively correlated with the increased survival of patients with several cancer types. TMEM52B might therefore be exploited as part of a novel strategy for cancer treatment. For example, full-length or partial fragments of TMEM52B may be candidates for an anti-cancer drug through gene therapy or administration of recombinant suppressor proteins or small molecule mimetics.

Consistent with our results, it has been recently reported that the downregulation of TMEM52B correlated with poor survival of renal cell carcinoma patients [10]. In contrast, TMEM52B is also associated with poor survival of gastric cancer patients, and reported to promote gastric cancer cell invasiveness and metastatic capacity [30]. Whether TMEM52B exerts positive or negative influences by cancer type or in a context-dependent manner needs to be further explored. Similarly, TMEM52B function and its precise molecular basis requires further exploration during both tumor development and in normal tissues.

TMEM52B appeared to be involved in the modulation of EGFR trafficking, based on our observation that TMEM52B suppression promoted EGFR endocytosis and enhanced its signaling. In general, the primary role of endocytosis is thought to terminate activation and signaling of receptor tyrosine kinases, including EGFR. However, internalization of activated EGFR has also been reported to enable specific signaling pathways from intracellular compartments, maintaining its signaling outcome [8]. As an example of the importance of trafficking regulation in tumorigenesis, Vav2, a Rho GTPase guanine nucleotide exchange factor, is known to regulate cell adhesion, spreading, motility, and proliferation in response to growth factor signaling. Vav2 is reported to delay EGFR internalization and degradation, thereby enhancing EGFR, ERK, and AKT phosphorylation [31]. Another example is the prolyl hydroxylase PHD3, a scaffolding protein associated with the endocytic adaptor Eps15. This protein promotes the internalization of EGFR and attenuates signaling to reduce cell proliferation and survival [32]. In contrast, reduction of SPRY2 (sprouty homolog 2) expression causes hyperactivation of PI3K/AKT signaling to drive prostate cancer cell proliferation and invasion by enhanced internalization of EGFR and sustained signaling at early endosomes in a PTEN-dependent manner [33]. Therefore, EGFR endocytosis can result in either positive or negative effects on signaling and tumorigenesis, suggesting conflicting or complex roles of EGFR trafficking.

On the other hand, we observed that TMEM52B suppression failed to substantially affect the interaction between c-Cbl and EGFR or ubiquitination of EGFR (Supplementary Fig. S7A). In addition, TMEM52B suppression did not substantially affect EGFR dimerization (Supplementary Fig. S7B). Together, these results suggest that TMEM52B suppression promotes cancer cell invasion and survival mainly through EGFR activation and internalization although we cannot completely rule out the possibility that other receptors are involved in TMEM52B function. Further studies should explore the precise molecular mechanisms of TMEM52B suppression-enhanced EGFR endocytosis and its sustained signaling.

E-cadherin exists in two forms: a membrane-tethered form and a soluble form. Proteolysis of intact E-cadherin leads to the release of an extracellular domain fragment of E-cadherin into the tumor microenvironment. This soluble E-cadherin is constitutively shed at low levels in normal epithelial cells, but significantly elevated in primary tumor sites and metastatic foci. Correlations between decreased survival and elevated soluble E-cadherin levels in serum, urine, and tumors have been reported [34, 35]. Soluble E-cadherin is reported to facilitate tumor cell invasion, proliferation, and survival [6, 36], thus exerting pro-oncogenic effects. We have demonstrated that TMEM52B suppression can downregulate E-cadherin both transcriptionally and via altered protein stability. Downregulation of E-cadherin results in reduced cell-cell adhesion, thereby promoting cell motility and activation of β-catenin, thus contributing to malignancy. In addition, the generation of soluble E-cadherin induced by TMEM52B suppression (likely through α-secretase, MMPs or ADAMs proteolysis) [6, 35], resulted in EGFR activation. Soluble E-cadherin is reported to activate EGFR family members and insulin-like growth factor receptor 1 (IGF-1R) on cancer cells and participate in the progression of various cancer types (autocrine signaling) [6]. It is possible that the soluble E-cadherin generated by TMEM52B suppression may exert its pro-tumorigenic activity directly to cancer cells and stroma cells in the tumor microenvironment, although it needs further investigation. For example, soluble E-cadherin is reported to activate KLRG1, an inhibitory receptor on immune effector cells, to attenuate the cytotoxic effect to cancer cells [6, 37]. Therefore, the downregulation of intact E-cadherin and the production of soluble E-cadherin, mediated by TMEM52B suppression, may contribute to the accelerated malignancy aggressiveness and tumor progression through both autocrine and paracrine mechanisms.

We observed that TMEM52B suppression enhanced EGFR phosphorylation in the absence of exogenous EGF as well as following treatment with exogenous EGF. In addition to the involvement of soluble E-cadherin, this may be attributed to the high expression of EGFR in the cancer cells used in this study, potentially because overexpression of EGFR and its oncogenic mutations can lead to spontaneous dimerization of EGFR, resulting in receptor activation [8]. Regulation of EGFR activation has also been attributed to its association with flotillin at the plasma membrane, in addition to its interaction with Ca^2+^/calmodulin complexes at the cytosolic juxtamembrane region of EGFR [8]. It is also possible that EGFR was phosphorylated by autocrine production of EGFR ligands.

EGFR is a target for an expanding class of anticancer therapies. EGFR-inhibiting agents such as EGFR tyrosine kinase inhibitors and EGFR-blocking antibodies are approved cancer treatments for a variety of cancers, including non-small-cell lung cancer, pancreatic, breast, and colorectal cancers. Due to innate or acquired resistance to these EGFR inhibitors, there remains an unmet need for further identification of potential molecular targets/pathways to enhance the development of novel effective therapeutics. It is possible that TMEM52B-based agents (e.g., TMEM52B functional-mimetics) may enhance the therapeutic benefit of EGFR-inhibiting agents, although this requires further investigation.

## Conclusions

In summary, we demonstrate that TMEM52B is a novel modulator of E-cadherin and EGFR activity. TMEM52B suppression reduces E-cadherin stability to produce soluble E-cadherin and enhances the activation and internalization of EGFR and its downstream signaling activity, leading to cancer cell invasion and survival. These results suggest that TMEM52B has tumor suppressor-like activity. Clinically, high TMEM52B expression correlated with increased survival among patients with varying types of cancer, implicating the use of TMEM52B as a potential prognostic biomarker. This study highlights the novel functions and underlying mechanisms of TMEM52B, suggesting its prominent role in tumor progression.

## Supplementary Information

**Additional file 1.**

## Data Availability

The datasets used and/or analyzed during the current study are available from the corresponding author on reasonable request.

## Abbreviations

CCLE: Cancer Cell Line Encyclopedia
CTC: circulating tumor cell
EGFR: epidermal growth factor receptor
EMT: epithelial-mesenchymal transition
ERK1/2: extracellular signal-regulated kinase 1/2
GFP: green fluorescent protein
HEK293E: human embryonic kidney 293E
JNK: c-Jun N-terminal kinase
qPCR: quantitative polymerase chain reaction
shRNA: short hairpin RNA
siRNA: small interfering RNA
TCGA: The Cancer Genome Atlas
