# Supplementary material for: TMEM52B suppression promotes cancer cell survival and invasion through modulating E-cadherin stability and EGFR activity
Source: J Exp Clin Cancer Res. 2021 Mar 1;40:58. doi: 10.1186/s13046-021-01828-7 (PMC7919321; doi:10.1186/s13046-021-01828-7)

TMEM52B suppression promotes cancer cell survival and invasion through modulating E-cadherin stability and EGFR activity

Yunhee Lee, Dongjoon Ko, Jung Hwa Yoon, Younghoon Lee, Semi Kim

Supplementary Figure Legends

Supplementary Figure S1. (A, B) HEK293E cells were transiently transfected with expression vectors for TMEM52B with a myc tag at the N-terminus for 48 h. (A) Overexpression of TMEM52B was analyzed by immunoblot using an anti-myc antibody. Of note, expression of isoform 2 was higher than that of isoform 1. (B) Flow cytometry analysis using PE-conjugated anti-myc antibody to show TMEM52B localization on cell membrane. (C, D) Cells were transiently transfected with siRNA or shRNA specific to TMEM52B for 48 h. Transfected cells were seeded onto 96-well plates at a density of 3×10^3 cells/well and incubated for 48 h. (C) Cell proliferation was determined by the colorimetric WST assay. (D) Proliferation rates were also estimated by the growth rate quotient (GRQ) as described in Materials and Methods. All determinations were performed in three independent experiments. Values represent mean ± SD. ns, not significant.

Supplementary Figure S2. Characterization of stable HCT-15 cells (TMEM52B-suppressed cells and control cells). (A) Immunoblot analysis revealed suppression of TMEM52B expression. Of note, EGFR phosphorylation was enhanced while E-cadherin was reduced in TMEM52B-suppressed cells, consistent with the results from transiently suppressed

cells (see Results section). (B) Anchorage-independent growth assay. Total numbers of colonies (>0.1 mm) were counted in six (×100) fields per well. Scale bar, 100 μm. (C) Invasion assay. (D) Analysis of cell survival under suspension culture conditions. All determinations were performed in three independent experiments. Values represent mean ± SD. *P < 0.05. (E) Tumor sections from Figure 2C were stained to detect E-cadherin and vimentin. Scale bar, 100 μm.

Supplementary Figure S3. (A) Cells were co-transfected with siRNA specific to TMEM52B and an AP-1 reporter plasmid for 48 h. AP-1 activity was determined by a reporter assay as described in Materials and Methods. (B) Cells were transfected with shRNA specific to TMEM52B (both isoforms) or siRNA specific to TMEM52B isoform 2 (but not isoform 1) for 48 h. Transfected cells were subjected to invasion assay or cell survival assay under suspension conditions as described in Figure 2A and B. All determinations were performed in three independent experiments. Values represent mean ± SD. *P < 0.05.

Supplementary Figure S4. (A) SW480sub cells were transfected with shRNA for 48 h followed by treatment with EGF (10 ng/ml) for the indicated times. EGFR, LAMP2, and DAPI were visualized; green for LAMP2, red for EGFR, and blue for DAPI staining. Co-localization of EGFR and LAMP2 was quantitated by calculating Pearson's correlation coefficient using ImageJ software. Values represent mean ± SD. *P < 0.05. (B) SW480sub cells transfected with TMEM52B-specific shRNA for 48 h were treated with EGF (20 ng/ml) for up to 30 min at 37°C to allow receptor internalization, or maintained at 4°C. Cells were stained with FITC-conjugated anti-EGFR and analyzed by flow cytometry to determine residual levels of cell surface EGFR. (C) SW480sub cells transfected with TMEM52B-specific shRNA for 42 h were

treated with Dynasore (160 µM) for 6 h prior to EGF treatment (10 ng/ml) for 5 min. Cells were stained with anti-EGFR (red) and anti-EEA1 (green). DAPI was used to visualize cell nuclei. Scale bar, 20 µm.

Supplementary Figure S5. (A-C) HCT-116 cells were transfected with TMEM52B-expressing vectors for 48 h. Invasion (A) and cell survival (B) assays were performed as described in Figure 2A and B, respectively. All determinations were performed in three independent experiments. Values represent mean ± SD. $^*P < 0.05$. (C) Transfected cells were lysed for immunoblot analysis. Anti-myc was used to detect myc-tagged TMEM52B.

Supplementary Figure S6. TMEM52B expression correlates with survival of cancer patients. (A-C) Kaplan–Meier analysis showed the probability of relapse-free survival from breast cancer patients data (KmPlotter, n = 1,764) (A), overall survival from lung cancer patients data (KmPlotter, n = 1,144) (B), and relapse-free survival from liver cancer patients data (KmPlotter, n = 316) (C) in relation to TMEM52B mRNA expression. TMEM52B expression was stratified as high vs. low according to an auto-select best cutoff value, and survival plots from previously published data sets were generated using http://kmplot.com (probe: 236646_at or 120939 for TMEM52B). (D) Kaplan-Meier analysis showed the probability of overall survival of kidney renal clear cell carcinoma (TCGA, Firehose Legacy) in relation to TMEM52B mRNA expression. All tumors with an mRNA expression profile (n = 534) were analyzed. High TMEM52B expression was defined by $Z > 1.5$. P values were calculated by the Logrank test.

Supplementary Figure S7. (A) TMEM52B suppression failed to substantially affect the interaction between c-Cbl and EGFR or ubiquitination of EGFR. SW480sub cells were transiently transfected with shRNA specific to TMEM52B for 48 h. Cells were lysed with co-immunoprecipitation buffer (10 mM Tris pH7.4, 150 mM NaCl, 0.56 mM EGTA, 1% Triton X-100, 0.5% NP-40, 5 mM N-ethylmaleimide) supplemented with protease inhibitor (Complete; Roche). Lysates were centrifuged for 20 min at 10,000*g*, and the resulting supernatant was precleared by incubating it with protein A/G-agarose for 2 h at 4°C. The precleared supernatant was immunoprecipitated using an anti-EGFR antibody (sc-120; Santa Cruz Biotechnology) at 4°C for 16 h. The protein complexes were collected by incubation with protein A/G-agarose for 2 h at 4°C and then washed four times with co-immunoprecipitation buffer. The protein complexes were eluted by boiling SDS sample buffer and analyzed by immunoblotting using anti-ubiquitin (P4D1; Santa Cruz Biotechnology), anti-c-Cbl (#2747; Cell Signaling), and anti-phospho-EGFR(Y1068) (#3777; Cell Signaling). (B) TMEM52B suppression did not substantially affect EGFR dimerization. HCT-15 cells were transiently transfected with shRNA specific to TMEM52B for 48 h. Transfected cells were treated with 1 mM bis(sulfosuccinimidyl)suberate (BS^3) for 2 h on ice prior to quenching with 10 mM Tris pH 7.5 for 15 min at room temperature. Cells were then lysed for immunoblot analysis using an anti-EGFR antibody (#4267; Cell Signaling). As a positive control for dimerization, transfected cells were treated with EGF (5 ng/ml) for 5 min before treatment with BS^3. Of note, unusually low percentage (4.5%) SDS-PAGE was used for immunoblotting to detect dimerized EGFR, resulting in the unusual detection of the monomer below the 150 kDa marker.

Supplementary Figure S1. Lee et al.

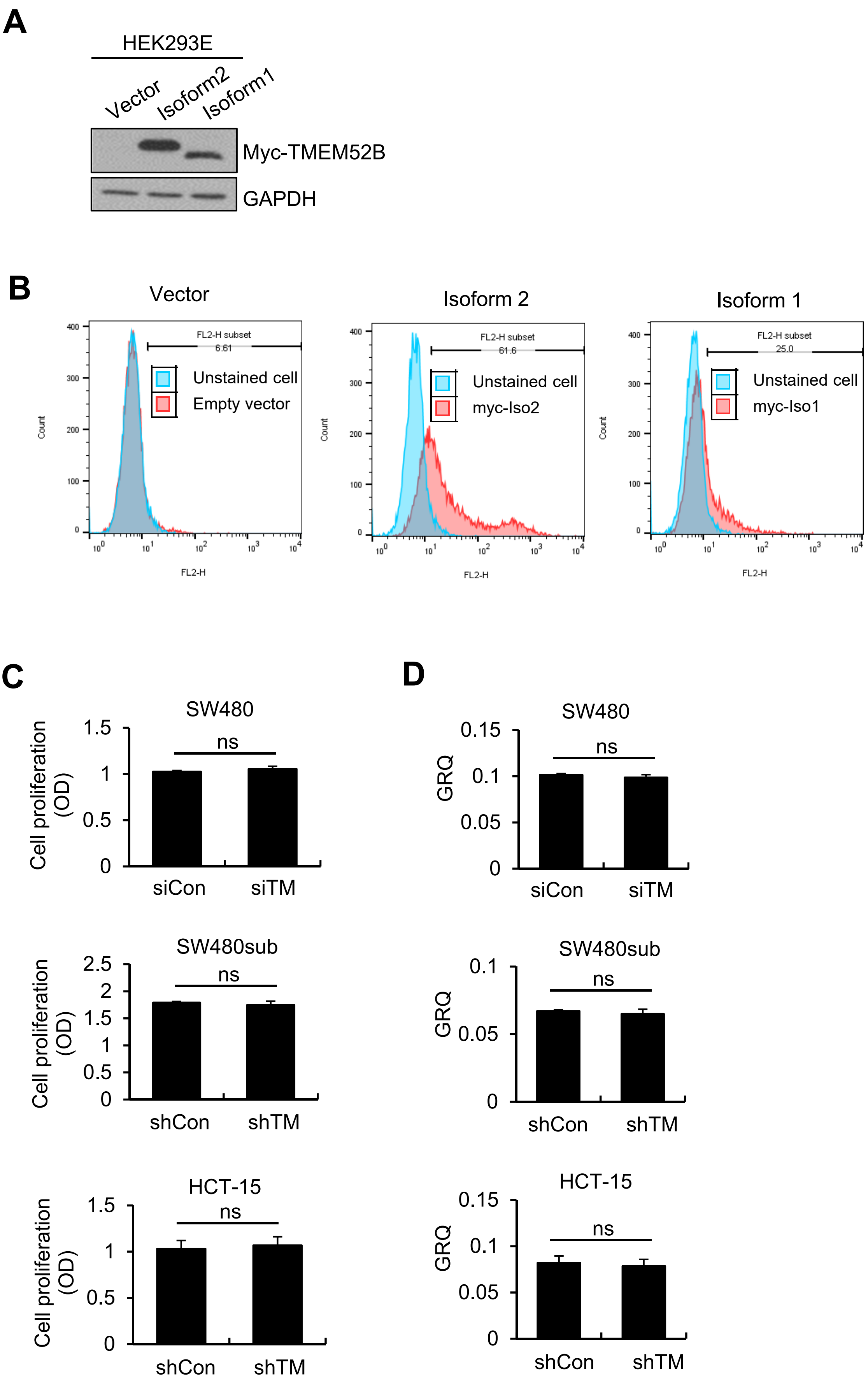

Supplementary Figure S2. Lee et al.

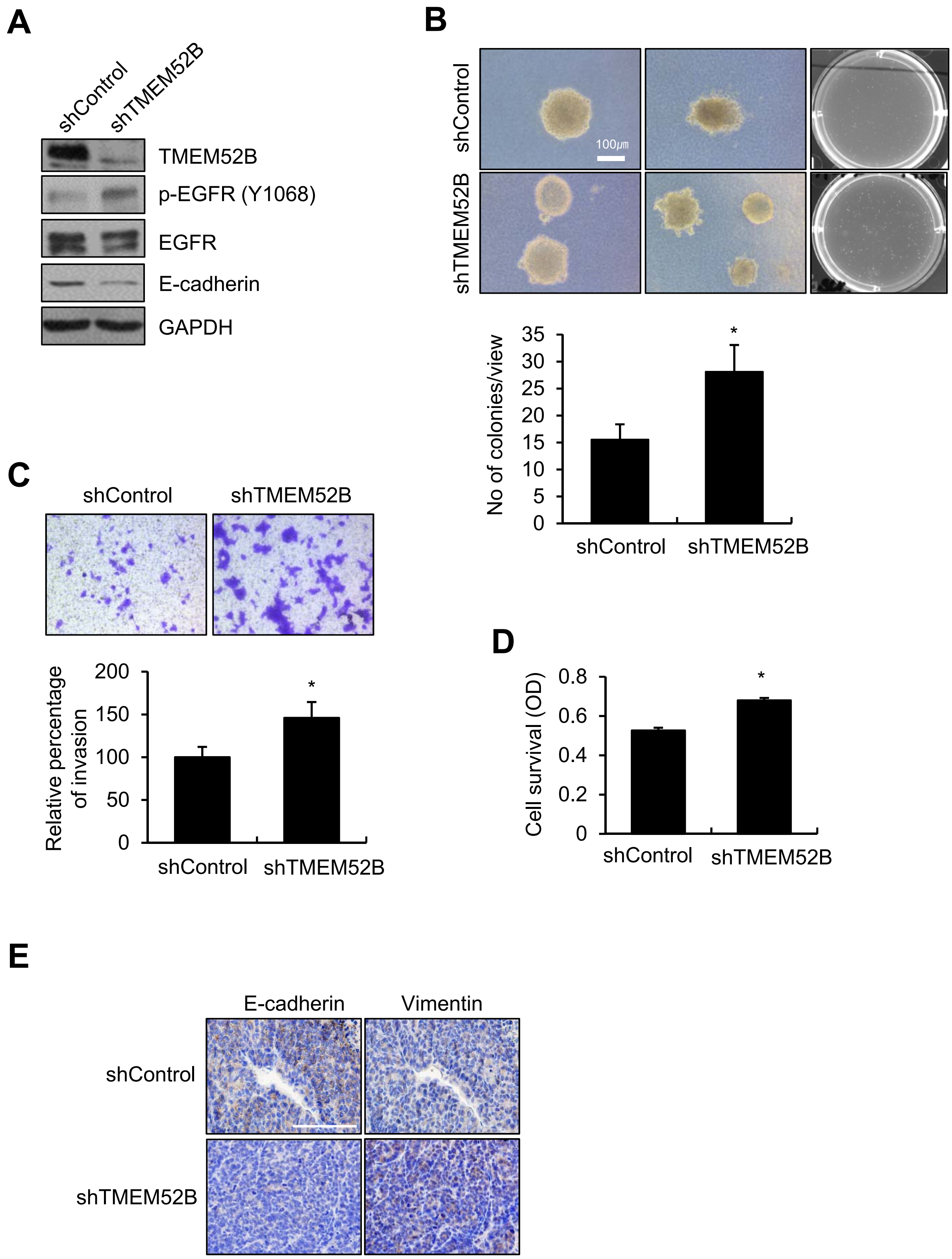

Supplementary Figure S3. Lee et al.

A

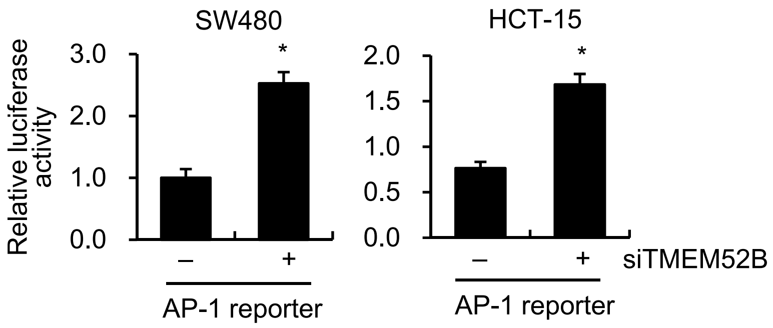

B

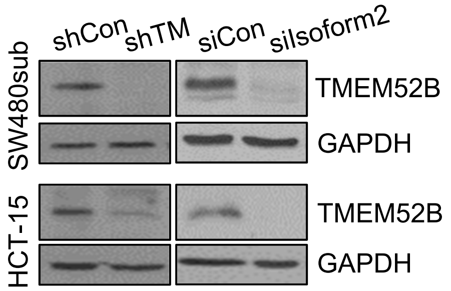

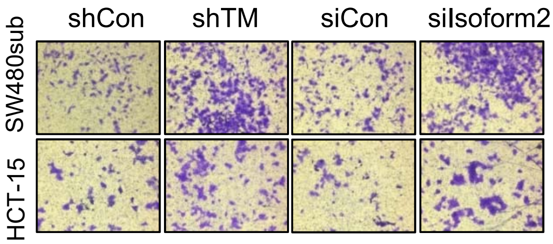

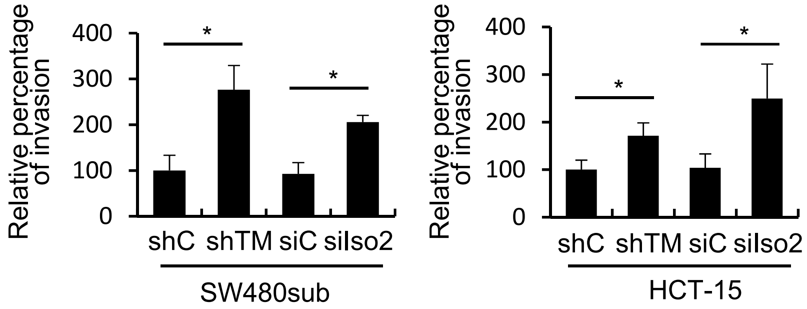

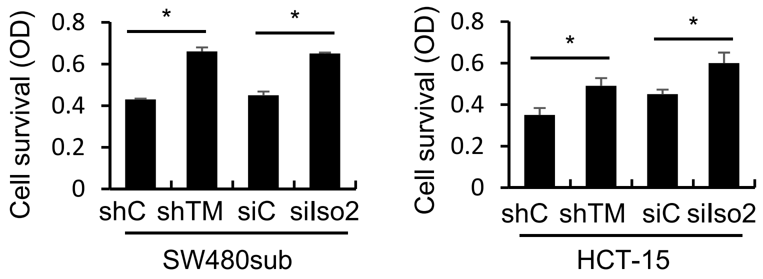

Supplementary Figure S4. Lee et al.

A

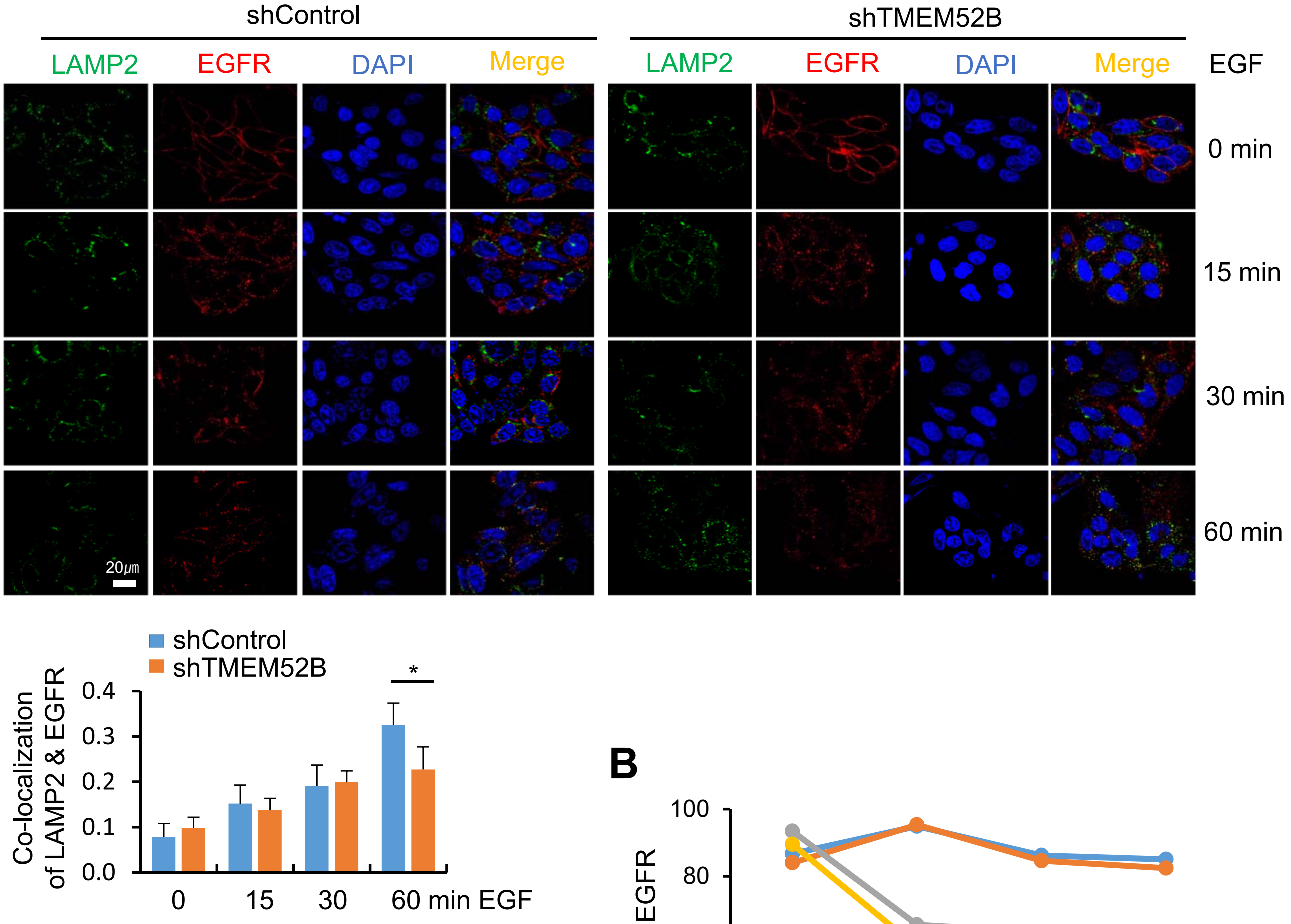

B

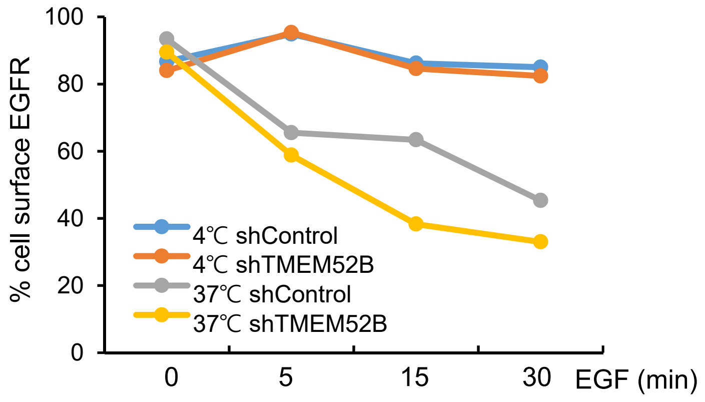

C

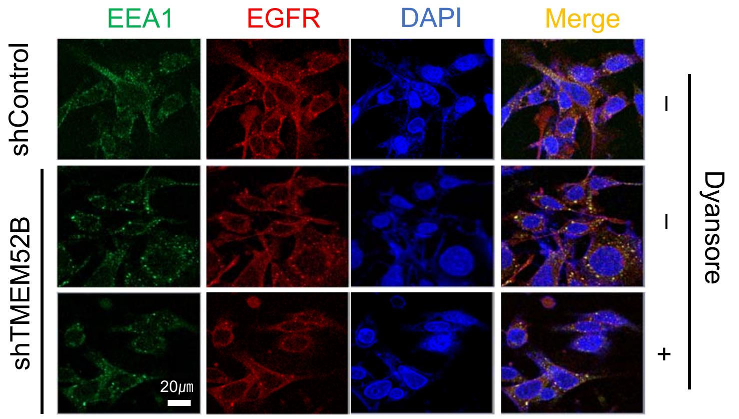

Supplementary Figure S5. Lee et al.

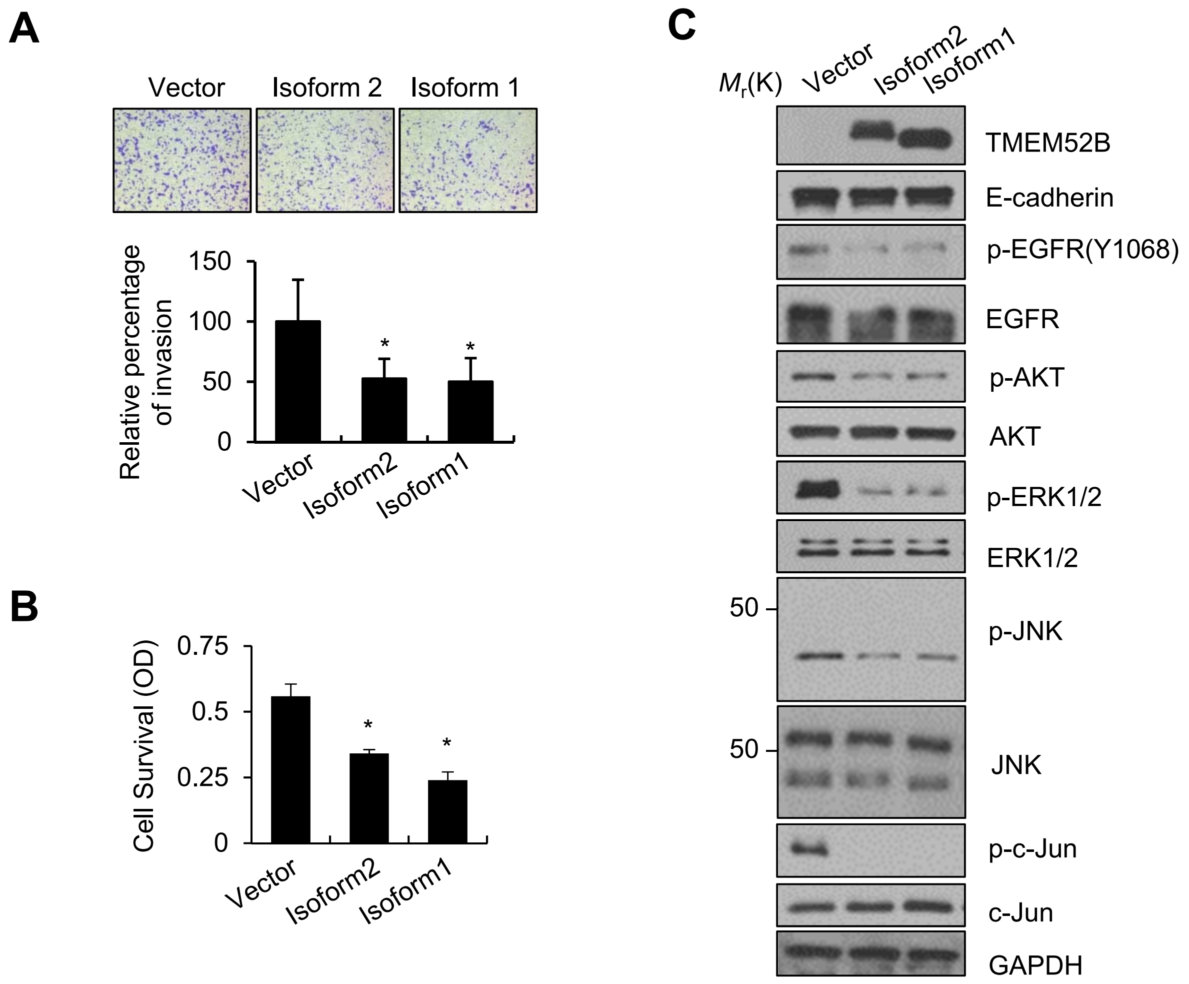

Supplementary Figure S6. Lee et al.

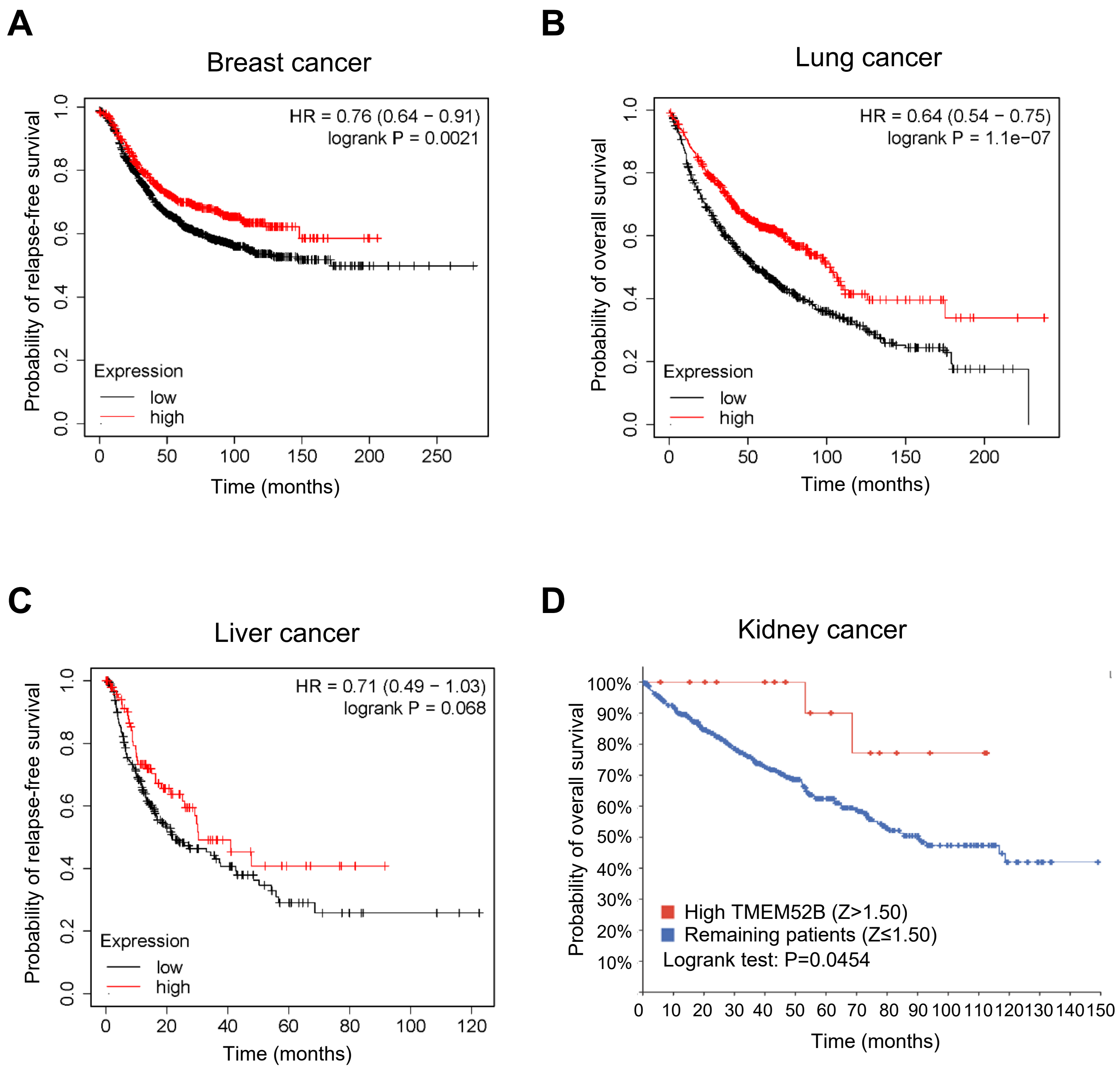

Supplementary Figure S7. Lee et al.

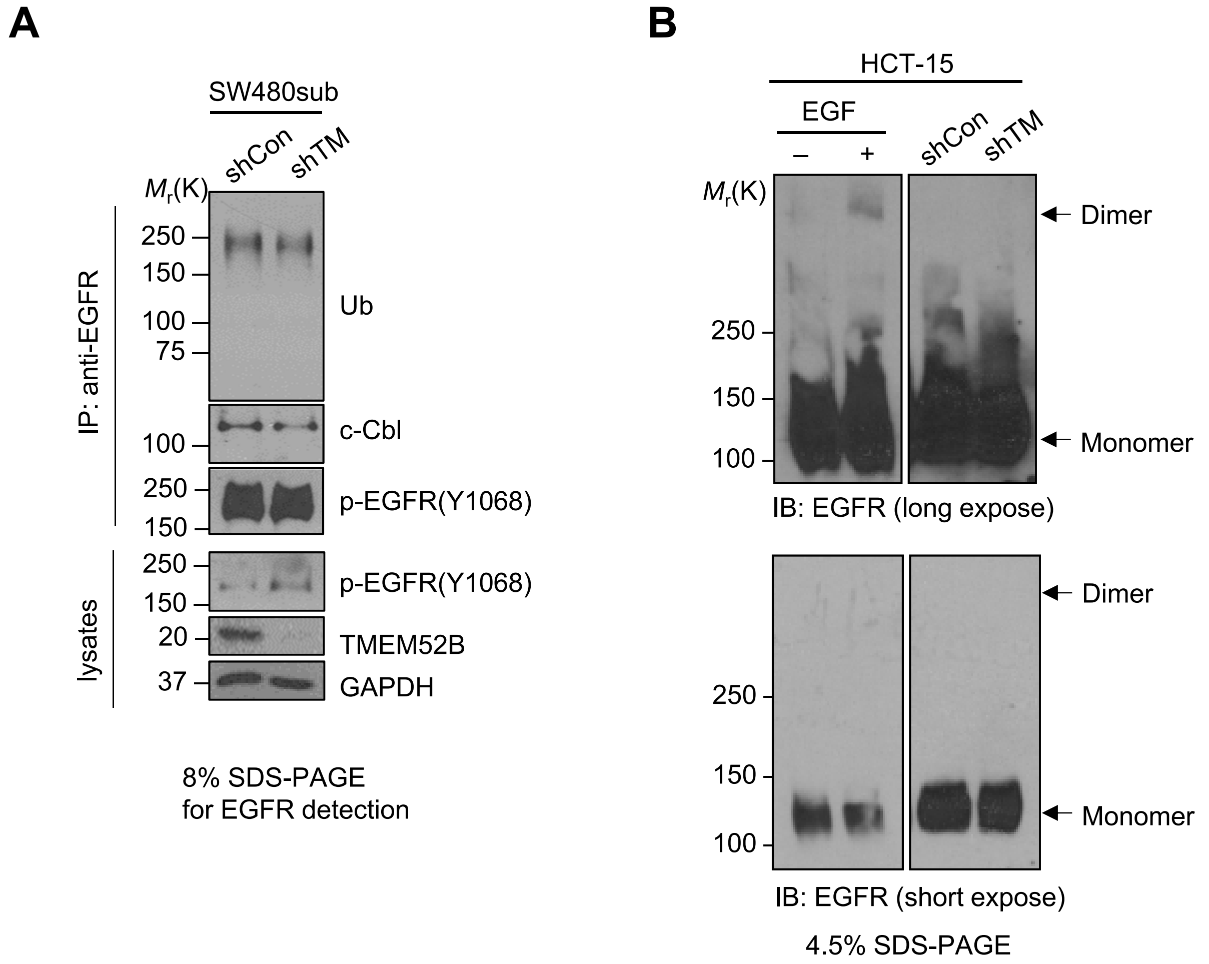

Supplement: Supplementary file 1 — Additional file 1. [file 13046_2021_1828_MOESM1_ESM.pdf]
